# Active diffusion and microtubule-based transport oppose myosin forces to position organelles in cells

**DOI:** 10.1038/ncomms11814

**Published:** 2016-06-02

**Authors:** Congping Lin, Martin Schuster, Sofia Cunha Guimaraes, Peter Ashwin, Michael Schrader, Jeremy Metz, Christian Hacker, Sarah Jane Gurr, Gero Steinberg

**Affiliations:** 1 School of Biosciences, University of Exeter, Stocker Road, Exeter EX4 4QD, UK; 2 Mathematics, University of Exeter, North Park Road, Exeter EX4 4QF, UK

## Abstract

Even distribution of peroxisomes (POs) and lipid droplets (LDs) is critical to their role in lipid and reactive oxygen species homeostasis. How even distribution is achieved remains elusive, but diffusive motion and directed motility may play a role. Here we show that in the fungus *Ustilago maydis* ∼95% of POs and LDs undergo diffusive motions. These movements require ATP and involve bidirectional early endosome motility, indicating that microtubule-associated membrane trafficking enhances diffusion of organelles. When early endosome transport is abolished, POs and LDs drift slowly towards the growing cell end. This pole-ward drift is facilitated by anterograde delivery of secretory cargo to the cell tip by myosin-5. Modelling reveals that microtubule-based directed transport and active diffusion support distribution, mobility and mixing of POs. In mammalian COS-7 cells, microtubules and F-actin also counteract each other to distribute POs. This highlights the importance of opposing cytoskeletal forces in organelle positioning in eukaryotes.

## Abstract (web summary)

The mechanisms underlying the positioning of eukaryotic organelles remain elusive. Here Lin *et al*. use imaging and a mathematical model to show that microtubule-based transport and active diffusion and actin-based polar drift act together to facilitate even distribution of peroxisomes in filamentous fungi.

## Graphical abstract

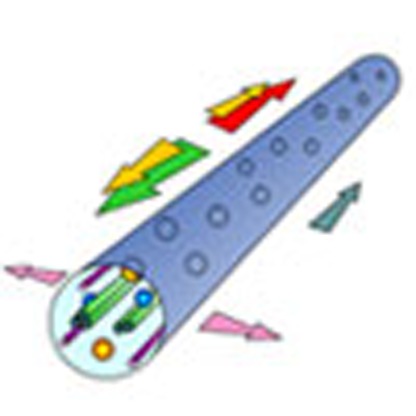

The ability of eukaryotic cells to position and distribute organelles appropriately is a general characteristic of cellular organization. Yet, the mechanisms underlying such distribution in a cell remain elusive. In particular, organelles that are involved in lipid homeostasis and fatty acid metabolism, such as peroxisomes (POs) and lipid droplets (LDs), are evenly positioned. This may support protection against oxidative stress^1^ and fosters dynamic interaction to transfer and distribute lipids, exchange metabolites or transduce signals^2^,^3^,^4^. Both organelles undergo directed transport (DT) and diffusive motion^5^,^6^,^7^. Diffusion (from Latin ‘diffundere'=spread out) describes the spread of molecules through random motion from regions of high to regions of low concentration. In liquids, larger particles behave in a similar manner, as first described for pollen grains in water^8^. This ‘Brownian motion' is a consequence of ceaseless bombardment by the thermal motion of neighbouring molecules, slowed by the viscosity of the surrounding liquid^9^,^10^. In the living cell, however, Brownian motion of organelles is largely restricted^11^. Instead, diffusive motion of organelles can be enhanced by ATP-dependent activity, such as molecular motors acting on the cytoskeleton^12^,^13^. To account for the mechanistic difference between thermal-induced and ATP-dependent random motion over short timescales, such diffusive behaviour of cellular structures is called ‘active diffusion' (AD)^14^,^15^.

The behaviour of POs and LDs in the filamentous fungi *U. maydis*, *Penicillium chrysogenum* and *Aspergillus nidulans* show similarities to mammalian cells. A small population of fungal LDs and POs undergo DT along microtubules (MTs)^16^,^17^,^18^, whereas the majority of the POs and LDs are scattered along the length of elongate hyphal cells, where they show short-range motions. DT of POs is also blocked when kinesin-3, or a Hook motor adapter on early endosomes (EEs) is deleted^16^,^18^,^19^. This is due to ‘hitchhiking' of POs on moving EEs^18^,^20^. Interestingly, in the absence of kinesin-3 and hook, POs cluster at the growing hyphal tip^16^,^18^,^19^,^20^. A similar clustering at the tip was described in dynamin mutants in *P. chrysogenum*^17^. The polar clustering of POs in Δ*dnm1* has been taken as an indication for the apical formation of these organelles^17^. Alternatively, unknown cytoplasmic forces may act on existing POs and ‘push' them to the hyphal tip, when MTs are disrupted.

Here we use the model fungus *U. maydis* to investigate the mechanism by which organelles are distributed in the hyphal cell. We show that F-actin and myosin-5 exert a polar drift (PD) force that moves POs and LDs to the growth region when MTs are absent. We further demonstrate that random motion of POs and LDs depends on MTs and involves bidirectional EE motility (energy-driven movement), which occurs along laterally bending MTs. In addition, we present a mathematical model, which predicts that AD and DT counteract actin-based PD to (i) distribute the POs, (ii) increase their mobility and (iii) to support their mixing in the cytoplasm. This suggests that the even distribution of organelles is an emergent property of these counteracting forces within the cell. Finally, we show that a similar balance between such cytoskeletal forces also distributes POs in mammalian COS-7 cells, suggesting that this may be a general principle for organelle distribution that is conserved from fungi to mammals.

## Results

### POs shift towards the hyphal tip in the absence of MTs

**Figure 1:**
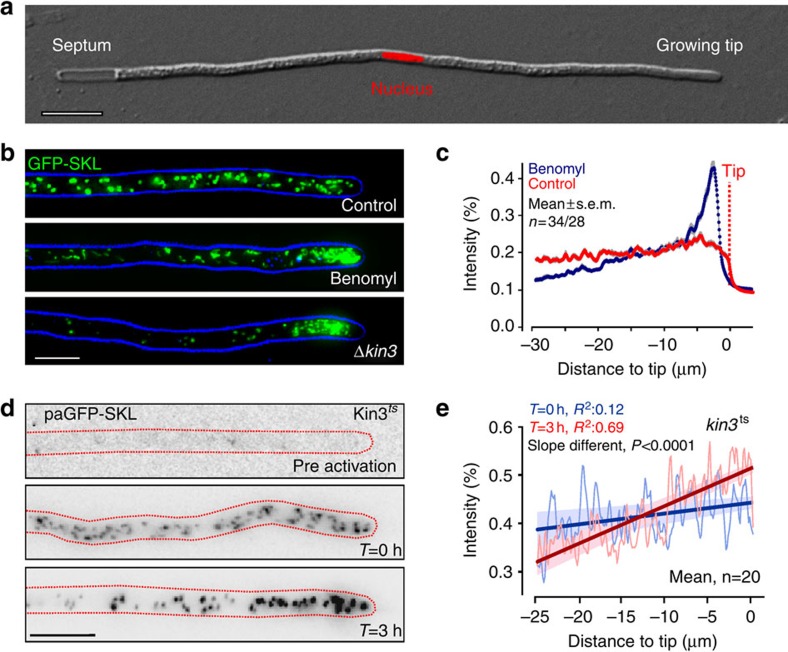
Peroxisomes migrate to the cell tip in the absence of MTs or kinesin-3. (**a**) Hyphal cell of *U. maydis*, expressing nuclear nls-RFP (nucleus). The cell expands at one cell end (growing tip), whereas forming a septum at the other end. The nucleus is positioned near the cell centre. Scale bar, 10 μm. (**b**) POs in untreated hyphal cells (Control) and cells treated for 5 h with 30 μM benomyl and kinesin-3-null mutants (Δ*kin3*). The organelles were labelled by GFP-SKL; the cell edge is indicated in blue. Image represents a maximum projection of a *z* axis image stack. Images were adjusted for brightness, contrast and gamma settings. Scale bar, 5 μm. (**c**) Fluorescence intensity profiles of GFP-SKL in hyphal cells treated for 5 h with the solvent DMSO (Control) or 30 μM benomyl. Each data point represents the mean±s.e.m. of measurements in 34 cells (Control) and 28 cells (Benomyl) from 2 experiments. The position of the cell tip is indicated. (**d**) Contrast-inverted images of temperature-sensitive kinesin-3^ts^ mutants that express paGFP-SKL. Before photoactivation, POs are not visible (pre-activation). After treatment with a 405-nm laser, fluorescent POs appear (*T*=0). After ∼3 h at restrictive temperature (32 °C), these photoactivated POs accumulate at the hyphal tip (*T*=3 h). Scale bars, 5 μm. (**e**) Fluorescence intensity profiles of GFP-SKL in temperature-sensitive *kin3*^ts^ cells at permissive temperature (blue profile) and 3 h at restrictive temperature (red profile). Each data point represents the average of measurements in 20 cells from 2 experiments. The shaded area corresponds to the 95% confidence interval for the fitting, which incorporated the s.e.m. of the experimental data. It is noteworthy that the slopes of the two curves are significantly different (*P*<0.0001, unpaired Student's *t*-test with Welch's correction).

In this study, we used the fungal model *U. maydis* to analyse the mechanism by which POs are distributed and mixed in a eukaryotic cell. *U. maydis* hyphae consist of a single elongate cell that expands at the growing tip and contains a central nucleus (Fig. 1a). We expressed the fluorescent PO marker GFP-SKL^18^ and found that POs were scattered along the hyphal cell (Fig. 1b, Control). At a given moment in time, the majority of the organelles showed short-range motion, whereas ∼5% of all POs underwent directed motility (4.54±2.78%, *n*=30 cells, 3 experiments, 3,539 POs, mean±s.d.; Supplementary Movie 1). This is in agreement with findings in mammalian CV1 cells^6^.

We showed recently that directed motility of POs depends on MTs^18^ (Supplementary Movie 2). We disrupted MTs using the fungal-specific MT inhibitor benomyl, which is effective in *U. maydis*^21^, and observed that POs form apical clusters in the hyphal cell (Fig. 1b,c). A similar result was obtained when genes for *U. maydis* kinesin-3 (*kin3*) or a hook adapter (*hok1*) were deleted (Fig. 1b Δ*kin3* and Supplementary Fig. 1a,b). Fungal Hook proteins link motors to EEs and, in their absence, EEs are immobile^19^,^22^, indicating that EEs are involved in PO distribution. Next we asked whether apical clustering is due to *de novo* formation of organelles at the tip or due to a pole-ward apical shift of existing POs. We tested this by introducing a photoactivatable PO reporter (paGFP-SKL) into conditional kinesin-3^*ts*^ mutants, which are impaired in MT-based transport at restrictive temperature. This reporter is not visible (Fig. 1d, pre-activation), until activated with a 405-nm laser pulse (Fig. 1d, *T*=0 h). Following 3 h at restrictive temperature (32 °C), when kinesin-3^*ts*^ is inactivated^23^, the previously photoactivated POs were found to concentrate at the hyphal tip (Fig. 1d, *T*=3 h after photoactivation and Fig. 1e). This result suggests that POs drift to the hyphal tip when MT-based transport is absent.

### Myosin-5 is responsible for a slow pole-ward drift of POs

**Figure 2:**
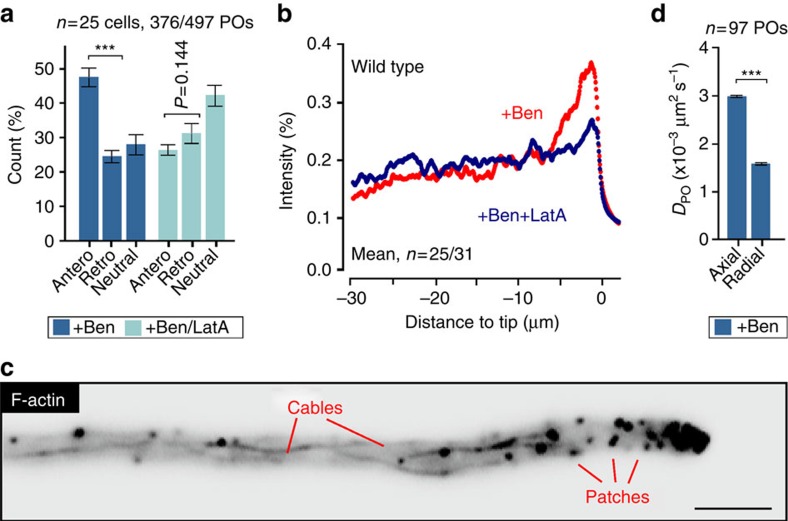
F-Actin supports pole-ward drift of POs. (**a**) Displacement of POs in the absence of MTs (+Ben) or MTs and F-actin (+Ben/LatA) over 10 s. Antero: towards the tip; Retro: away from the tip. Less anterograde displacement occurs when MTs and F-actin are disassembled. Mean±s.e.m. and sample size *n* is shown. ***Significance at *P*<0.0001, unpaired Student's *t*-test with Welch's correction. (**b**) Average fluorescence intensity profiles of GFP-SKL in cells treated for 5 h with benomyl (+Ben, red) or benomyl and latrunculin A (+Ben/LatA, blue). It is noteworthy that the absence of F-actin prevents formation of an apical PO cluster. Each data point represents mean of measurements in 25 (+Ben) or 31 (+Ben/LatA) cells from two experiments. (**c**) Contrast-inverted image showing F-actin, labelled with LifeAct-GFP, in a hyphal cell. The cell contains F-actin patches and long F-actin cables. Scale bar, 3 μm. (**d**) Axial and lateral diffusion coefficient of POs (*D*_PO_), derived from MSD analysis, in hyphal cells treated with benomyl. In the absence of MTs, diffusion is extended along the axis of the cell. Best fitted *D*_PO_±s.e.m. from linear fitting. ***Statistical significance at *P*<0.0001, F-test.

Next, we set out to gain insight into the mechanism for PD of POs in the absence of MTs. First, we attempted to determine the velocity of this PD in cells treated with benomyl. However, drift velocity was too low to be detected in kymographs. We therefore estimated the drift velocity using the average distribution curves of Δ*kin3* mutants (see Supplementary Methods). This revealed an estimated average PD velocity of 0.00044 μm s^−1^, a value ∼4,000 times lower than motor-based transport recorded in fungi^16^,^24^,^25^. We asked whether this PD is due to F-actin-related processes that are masked when MTs are present. To test this idea, we disrupted MTs and investigated the displacement of individual POs over a period of 10 s, relative to the position of the hyphal tip. When MTs were disassembled, significantly more POs moved to the tip (Fig. 2a, anterograde; *P*<0.0001; unpaired Student's *t*-test with Welch's correction). When MTs and F-actin were depolymerized simultaneously, using benomyl and latrunculinA, which depolymerises F-actin in *U. maydis*^21^, POs did not move towards the hyphal tip (Fig. 2a, +Ben/LatA; *P*=0.144). In addition, no PO clustering occurred when MTs and F-actin were disassembled (Fig. 2b, +Ben/LatA). The same result was found in temperature-sensitive *kin3*^ts^ mutants that were shifted to restrictive temperature in the presence of the actin inhibitor latrunculinA (Supplementary Fig. 1c). These results strongly suggest that F-actin-based processes drive POs towards the growing cell tip when MTs are disrupted.

**Figure 3:**
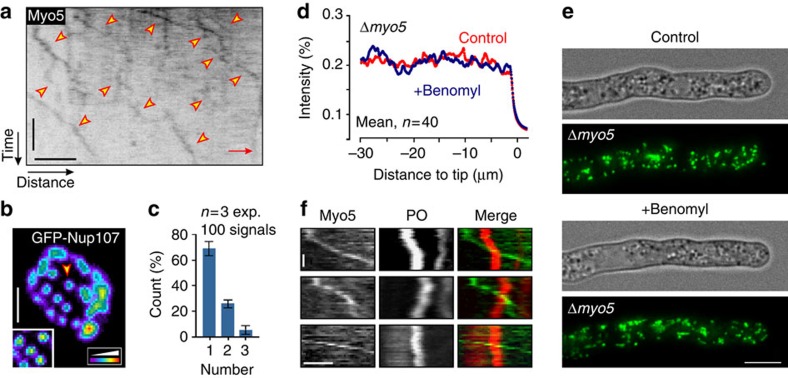
Anterograde motility of myosin-5 drives pole-ward drift of POs. (**a**) Contrast-inverted kymograph of GFP_3_-Myo5 motility (arrowheads). The position of the growing hyphal tip is indicated (Tip). Scale bars, 6 s (vertical) and 2 μm (horizontal). See Supplementary Movie 3. (**b**) False-coloured image of GFP-Nup107 in nuclear pores of *U. maydis*. Arrow marks individual nuclear pores, which contain 16 GFP-Nup105 molecules^30^. Scale bar, 1 μm. (**c**) Bar chart showing estimated myosin-5 numbers in moving GFP_3_Myo5 signals. Estimation is based on a comparison of fluorescent intensity with GFP-Nup107 as internal calibration standard. Mean±s.e.m. and sample size *n* is shown. It is noteworthy that myosin-5 is assumed to contain two heavy chains^62^, encoded by the *myo5* gene in *U. maydis*^29^. (**d**) Average fluorescence intensity profiles of GFP-SKL in Δ*myo5* mutants, treated for 5 h with the solvent DMSO (Control, red) or benomyl (blue). In the absence of Myo5, depolymerization of MTs did not induce apical PO clustering. (**e**) PO distribution in Δ*myo5* cells, treated with DMSO (Control) or benomyl. In the absence of Myo5, depolymerization of MTs did not induce apical PO clustering. Images were adjusted for brightness, contrast and gamma settings. Scale bar, 5 μm. (**f**) Kymographs showing three examples of GFP_3_-Myo5 (green) passing mCherry-containing POs (red). No co-migration of both was observed. Scale bars, 1 s (vertical) and 1 μm (horizontal).

*U. maydis* cells contain long actin cables (Fig. 2c)^21^, suggesting that F-actin-based motor activity, along the axis of the cell, might drive POs towards the hyphal tip. We tested this idea by disrupting MTs with benomyl and then determining the extent of short-range motion of individual organelles, given as the diffusion coefficient *D*_PO_, in the axial and radial direction within hyphal cells. Indeed, short-range walks of POs were significantly extended along the axis of the cell (*P*<0.0001, F-test; Fig. 2d). We therefore hypothesized that transport along F-actin cables could exert force on POs. If such a force is unidirectional, it could account for the F-actin-dependent PD of POs in benomyl-treated cells. In fungi, actin-associated myosin-5 is thought to deliver secretory vesicles towards the growing hyphal tip^26^,^27^,^28^. We therefore tested for such anterograde transport by observing a fusion of 3 × green fluorescent protein (GFP) to the native *myo5* gene, which encodes the heavy chain of class V myosin in *U. maydis*^29^. Most myosin-5 moved along the cell periphery towards the hyphal tip (89.6±1.3%, *n*=3 experiments, 135 signals, 20 cells; mean±s.d.; Fig. 3a and Supplementary Movie 3). Next we asked whether the fluorescent signals represent single motors. GFP_3_Myo5-expressing strain contained a triple-GFP tag fused to the endogenous *myo5*. Consequently, all myosin-5 motor molecules in the cell carry two GFP_3_ tags (six GFPs). We measured the fluorescence intensity of the moving GFP_3_Myo5 signals and compared them with fluorescent nuclear pores, where each pore contains 16 GFP-Nup107 nucleoporin fusion proteins (Fig. 3b; individual pore highlighted by arrowhead and in inset). This internal calibration was used previously to determine motor numbers in living cells of *U. maydis*^23^,^30^. This analysis revealed that ∼70% of the moving GFP_3_Myo5 signal represent single myosin-5 motors (Fig. 3c).

The constant movement of GFP_3_Myo5 towards the cell tip is consistent with the notion that myosin-5 movement to the growth region provides the force for PD of POs. We tested this prediction directly by observing POs in Δ*myo5* mutants^29^ and found that POs are evenly distributed along the Myo5-deficient cells (Fig. 3d,e, Control). When MTs were disrupted, however, POs did not shift to the growing tip (Fig. 3d,e, Benomyl). This result supports the idea that myosin-5 activity drifts POs towards the cell pole when MT-based forces are abolished. To investigate whether GFP_3_Myo5 directly transports PO, we co-visualized the motor and mCherry-SKL in hyphal cells. We did not find co-migration of the motor and POs (Fig. 3f). Finally, we measured the velocity of GFP_3_-Myo5 movements. With 1.29±0.51 μm s^−1^ (*n*=162, 3 experiments; mean±s.d.), this velocity exceeds the estimated drift velocity ∼3,000 times, again making it unlikely that the motor moves POs directly. As we have no evidence for a direct role of myosin-5 in the slow apical drift of POs, we suggest that continuous myosin-5 transport of secretory cargo towards the hyphal tip generates an apical flux that drives POs to the apex when MT-based transport processes are impaired.

Our data indicate that actin-based traffic exerts an intrinsic and unspecific force on organelles. If correct, other organelles should also undergo PD when MTs are disrupted. We therefore observed LDs, labelled with the putative methyltransferase Erg6-GFP^18^. Similar to POs, LDs were scattered along the hyphal cell (Supplementary Fig. 2a). Most LDs displayed short-range motion, whereas a small proportion underwent DT over longer distances (4.32±4.05%, *n*=90, 30 cells, 3 experiments, 1,923 LDs; mean±s.d.), therefore behaving in a manner similar to POs. When MTs were disrupted, or EE motility blocked in *hok1* mutants, LDs also clustered at the hyphal tip (Supplementary Fig. 2b–d). This aggregation was abolished when F-actin was disrupted (Supplementary Fig. 2d). We conclude that F-actin based transport also exerts a pole-ward force on LDs, which is overcome by MT-associated EE motility. This supports a model whereby F-actin-based and myosin-driven motility exerts a nonspecific pole-wards force on organelles.

### EEs support AD of POs

**Figure 4:**
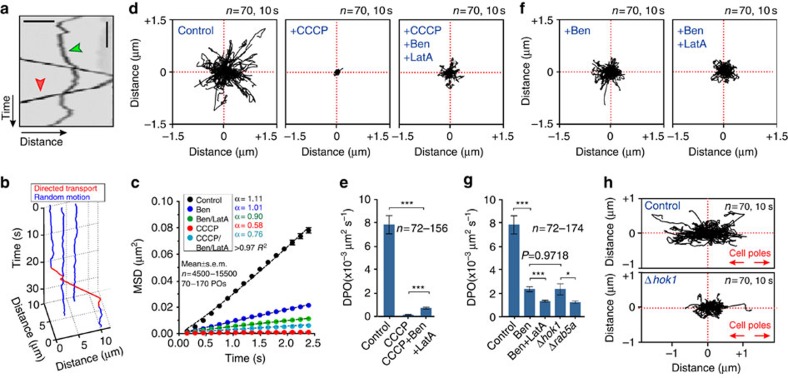
Random motion of POs depends largely on MT-mediated EE motility. (**a**) Contrast-inverted kymograph showing directed motility of POs (red arrowhead) and random motion (green arrowhead). Scale bars, 3 s (vertical) and 2 μm (horizontal). See Supplementary Movie 1. (**b**) Switch between random motion (blue) and directed PO motility (red). See Supplementary Movies 1 and 2. (**c**) MSD analysis of random PO motion in the presence of DMSO (Control, black), benomyl (Ben, blue), a combination of benomyl and latrunculin A (Ben/LatA, green) or CCCP (red curve). Mean±s.e.m. is shown; bars based on *n*=4,500–15,500 measurements of 72–156 POs. (**d**) Random motions of POs in *U. maydis*, treated with DMSO (Control), CCCP (+CCCP) or a combination of CCCP, benomyl and latrunculin A (+CCCP, +Ben, +LatA). Plots show 70 POs over 10 s, starting at the centre. Disrupting the cytoskeleton slightly restores PO diffusion. See Supplementary Movie 4. (**e**) Diffusion coefficients (*D*_PO_), derived from MSD analysis, in the presence of DMSO (Control), benomyl (Ben), CCCP and a combination of CCCP, benomyl and latrunculin A (CCCP, +Ben, +LatA). Mean±s.e.m. is shown; bars based on 72–156 POs. ***Statistical significance at *P*<0.0001, unpaired Student's *t*-test with Welch's correction. (**f**) Random motions of POs in *U. maydis*, treated with DMSO (Control), benomyl (+Ben), a combination of benomyl and latrunculin A (+Ben, +LatA). Plots show 70 POs over 10 s, starting at the centre. Disrupting MTs has a dramatic effect on random PO motion. See Supplementary Movie 4. (**g**) Diffusion coefficients (*D*_PO_), in the presence of DMSO (Control), benomyl (Ben), a combination of benomyl and latrunculin A (Ben+LatA), or in the absence of the endosomal motor adapter Hok1 (Δ*hok1*) or the endosomal GTPase Rab5a (Δ*rab5a*). Mean±s.e.m. is shown; bars based on 72–174 POs. ***Statistical significance at *P*<0.0001; *Statistical significance at *P*=0.0131; no significant difference between benomyl-treated cells and Δ*hok1*, *P*=0.9718, unpaired Student's *T*-test with Welch's correction. (**h**) Random motion of POs in relation to the hyphal orientation in untreated cells (control) and *hok1*-null mutants (Δ*Hok1*). Horizontal direction corresponds to the cell axis (red arrows direct to cell poles). Plots show 70 POs over 10 s, starting at the centre.

In *U. maydis*, ∼5% of the POs displayed directed long-range motility along MTs (Fig. 4a, red arrowhead), whereas the majority of the organelles showed short-range motions (Fig. 4a, green arrowhead; Supplementary Movie 2). POs were found to switch between both states (Fig. 4b). Short-range motion included occasional directed displacements (<2 μm; Supplementary Fig. 1d; yellow arrowheads), which were slower than long-range DT (0.20±0.01 μm s^−1^, *n*=72; *P*<0.0001, unpaired Student's *t*-test with Welch's correction; mean±s.d.). We asked whether short-range motion show characteristics of random diffusion by analysing individual PO motions, using mean square displacement (MSD) analysis. This is a powerful tool that allows one to distinguish directed motility from various types of random motion^15^,^31^,^32^. The obtained curve of MSD against time *t* can be fitted to *t*^*α*^ where the exponent *α*∼1 indicates diffusive and random behaviour and *α*∼2 indicates continuous DT. We found that MSD curves of PO short-range motions increased approximately linearly, with *α*=1.11 over 2.5 s (Fig. 4c, Control) and *α*=0.86 over 20 s (Supplementary Fig. 1e). As the MSD of the short-range motions of POs increases approximately linear with time, we refer to this motion as diffusive and random.

Work in mammalian cells has shown that random PO motions are ATP dependent^7^. We inhibited enzymatic activity in *U. maydis* with carbonyl cyanide *m*-chlorophenyl hydrazone (CCCP). This drug impairs cell respiration, thereby reducing cellular ATP levels^33^, and was used previously to investigate intracellular motility in *U. maydis*^28^ and random PO motion in mammalian cells^34^. Under these conditions, random motions were drastically reduced (Fig. 4d, Control and +CCCP, and Supplementary Movie 4). Thus, we conclude that random walking of POs in *U. maydis* requires ATP.

We determined the diffusion coefficient (*D*_PO_) of each treatment from MSD curves. This revealed that extend of random PO walking fell by >98% when CCCP was added (Fig. 4e). We considered it possible that this effect is a consequence of increase of viscosity, or ‘stiffening', of the cytoplasm at low ATP levels, as has been described as being due to the cytoskeletal structure in other systems^35^. To test this, we simultaneously treated cells with CCCP and the cytoskeleton inhibitors benomyl and latrunculin A. Indeed, by removing the cytoskeleton, PO diffusion was restored slightly (Fig. 4d, +Ben, +LatA and +CCCP), with a *D*_PO_ that reached ∼9.5% of control (Fig. 4e). Thus, we conclude that restriction by cytoskeletal elements plays a relatively minor part in the inhibition of random PO motion under low ATP levels. This suggests that enzymatic activity supports AD of POs.

We next tested whether cytoskeleton-associated processes participate in AD of POs. Indeed, we found random motion of POs was drastically reduced when MTs are depolymerized (Fig. 4f,g +Ben and Supplementary Movie 4). Disassembling F-actin and MTs simultaneously further decreased random PO motion (Fig. 4f,g +Ben/+LatA and Supplementary Movie 4). However, these data show that MT-associated processes have the greatest impact on random PO motion. We tested whether LDs also undergo AD. Indeed, their random motions are also consistent with diffusion (Supplementary Fig. 3a,b, *α*=0.911), inhibited by CCCP and depend on the presence of MTs (Supplementary Fig. 3a–c). We conclude that MT-associated enzymatic activity underlies the diffusive random motions of both POs and LDs.

To determine which MT-based process enhances random walking of POs in *U. maydis*, we observed random motion of POs in the proximity of GFP-labelled MTs. We found that POs, when located close to MTs, accelerated into short axial motions (Supplementary Movie 5; Supplementary Fig. 1d and Fig. 4h, Control). This suggests that membrane trafficking along MTs could enhance PO diffusive motions. In hyphal cells of *U. maydis*, EEs constantly move along MTs in a bidirectional manner^24^,^30^. We therefore tested whether EEs motility enhance PO random motion. We made use of Δ*hok1* mutants, in which motors are not bound to EEs and thus their motility is blocked^22^. We found that random motion of POs was significantly impaired in Δ*hok1* mutants (Fig. 4h, Δ*hok1*). Indeed, the *D*_PO_ in Δ*hok1* mutant was indistinguishable from that measured in the absence of MTs (Fig. 4g; *P*=0.9718, unpaired Student's *t*-test with Welch's correction). We confirmed a role of EEs in random motion by monitoring POs in mutants deleted in the small EE-associated GTPase Rab5a, which is required for EE motility in *U. maydis*^36^. Consistent with a role of EEs in diffusive PO motion, the *D*_PO_ was drastically reduced (Fig. 4g). Finally, we investigated a short-term reaction of cells to inhibition of EE motility in temperature-sensitive *kin3*^ts^ mutants. After 30 min at restrictive temperature, EE motility was fully blocked due to deactivation of kinesin-3 (ref. 23), which resulted in a reduction in random motion of POs (Supplementary Fig. 4a,b, 32 °C). Collectively, our data strongly indicate that MT-based EE transport is responsible for the AD of POs.

### Evaluation of mechanisms involved in PO distribution

**Figure 5:**
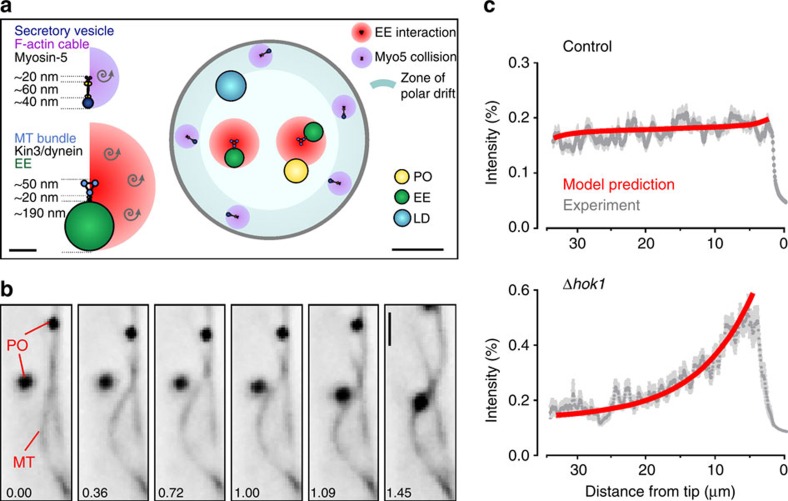
Mathematical modelling of PO behaviour in hyphal cells. (**a**) Diagrams showing spatial dimensions and arrangement of POs, EEs, MTs and F-actin in a hyphal cell cross-section. MT numbers in bundles, their diameters and the bridging distance provided by kinesin motors were obtained from published literature (see main text and Table 1). The average size of EEs and POs was measured in electron micrographs. Cells contain peripheral F-actin cables (see Supplementary Fig. 5c) and Myo5 streaming along F-actin cables occurs at the periphery of the cell (see Supplementary Movie 3); thus, pole-ward drift forces are considered most effective at the cell periphery (blue ring). Scale bars, 0.1 and 0.5 μm. (**b**) Image series showing lateral bending of a MT, which results in contact with a PO. Both structures are labelled with GFP, but due to their appearance are easily identifiable. Time in seconds given in lower left corner; Scale bar, 1 μm. See Supplementary Movie 6. (**c**) Predicted distribution of POs, shown as fluorescent intensity of GFP-SKL over the apical 30 μm of a hyphal cell. The predicted data fit the experimentally determined distribution curve (Control, grey curve; mean±s.e.m.). When EE-based AD and DT are removed from the modelling, the distribution curve matches the distribution of POs in a *hok1*-null mutant (Δ*hok1*, grey curve), where EE motility is stopped^22^.

We have demonstrated that actin-based PD forces are opposed by MT-associated processes and have identified bidirectional EE motility as the underlying mechanism for AD of POs. However, we have shown recently that EEs are also responsible for DT of POs, as they move the organelles over long distances along MTs^18^. Thus, both AD and DT of POs in *U. maydis* involve the same transport machinery. To better understand the relative contribution of each process to PO distribution and mobility, we reconstructed the spatial architecture of the hyphal cell (Fig. 5a). We used published data of numbers and dimensions of MTs in *U. maydis*, dimension of LDs from *Saccharomyces cerevisiae* (Table 1) and determined the size of POs and EEs in electron micrographs (Supplementary Fig. 5a,b). We accounted for the peripheral localization of F-actin cables^37^ (Supplementary Fig. 5c), where most GFP_3_-Myo5 movements occur (Supplementary Movie 3). In contrast, MTs located more centrally within the hyphal cell (Supplementary Fig. 5c and Fig. 5a). However, MTs in *U. maydis* have been shown to undergo motor-driven bending of MTs^38^,^39^,^40^, which most probably increases the chance of interaction between peripheral organelles and MT-associated EEs (Fig. 5b and Supplementary Movie 6). In fruit flies, such behaviour of MTs drives PO motions^31^. We tested for such a mechanism in *U. maydis*, but co-observation of POs and MTs revealed only rare co-motility of POs and bending MTs (1.71±1.30%,*n*=3 experiments, 30 cells; mean±s.d.). We therefore considered this mechanism of minor importance in *U. maydis*.

### A mathematical model to describe PO organization

Next, we developed a partial differential equation model for three populations of POs along the axis of a single cell (for details on modelling and used values, see Supplementary Tables 1 and 2, and Supplementary Methods). Two of the populations represent POs undergoing long-distance motility on MTs in anterograde or retrograde direction (DT with a finite average persistency of ∼6.5 μm in each direction), respectively. The third population represents POs undergoing short-range and random motions within the cytoplasm, driven by a combination of a slow polar actin-based drift and AD. The model includes transitions between diffusive PO motions and directed PO transport, as well as reversals of DT. As the EEs, which drive DT of POs^18^, are not observed to typically fall off at the end of MTs or form clusters at MT tips^30^, we assume that directly transported POs immediately reverse direction on reaching the ends of the domain. We validated the model by comparing the predicted PO distributions, indicated by fluorescent intensity profiles, with experimental results from hyphal cells. Our model predicted accurately the PO distribution that we observed both in control cells (Fig. 5c, Control) and in *hok1*-null mutants, in which EEs no longer move (Fig. 5c, Δ*hok1*). Our model is therefore a valuable mathematical tool for dissecting the relative contribution of AD and DT to PO mobility and distribution in the cell.

### AD and DT cooperate to mix and distribute POs

**Figure 6:**
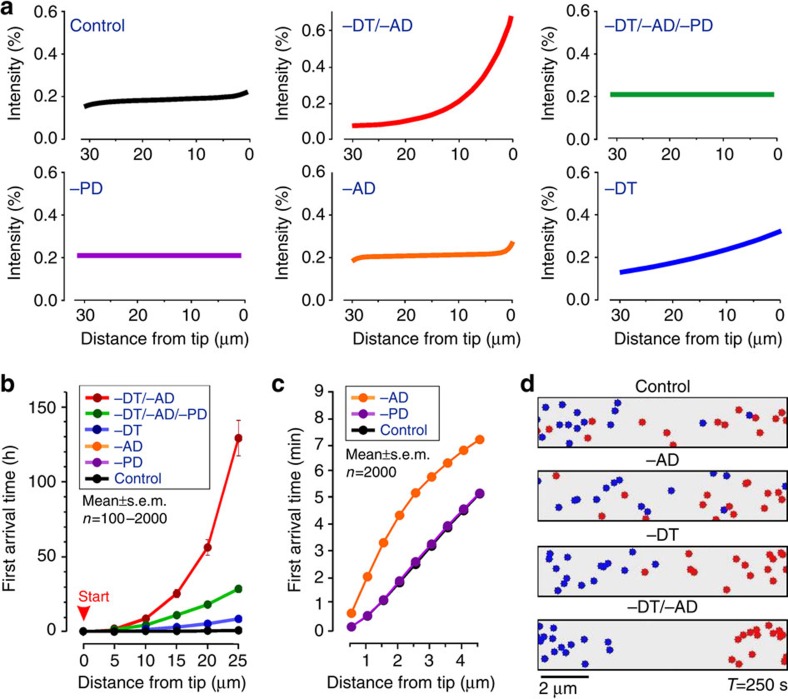
Modelling of PO distribution and mixing. (**a**) Predicted PO distribution, given as intensity profile along the apical 30 μm of hyphal cells. −DT, no directed transport; −AD, no AD; −PD, no PD; and combinations. POs are evenly distributed in control cells (upper left). In the absence of AD and DT, POs cluster at the hyphal tip (upper middle, −DT/−AD). With no PD, no clustering occurs (upper right; −DT/−AD/−PD). MT-associated processes do balance, as no clustering occurs when only PD is excluded (lower left; −PD). In the absence of AD, POs are still evenly distributed (lower middle; −AD). When active transport is absent, slight aggregation of POs towards the growing tip is predicted (lower right; −DT). Polar clustering is stronger when AD and DT are excluded (upper middle, compare with lower right), suggesting that both processes cooperate in distributing POs. Colour coding matches **b**,**c**. (**b**) Predicted PO mobility over long distance under various conditions (−AD, no AD; −DT, no DT; −PD, no PD; and combinations of these). The average time required for a PO to move from the tip (indicated by START) to sub-apical regions is indicated. Data points are provided as mean±s.e.m., *n*=100–2,000 simulations. Mobility is drastically impaired when AD and DT are excluded. It is also noteworthy that curves for ‘noPD' and ‘no AD' are covered by the control curve. (**c**) Predicted PO mobility over short distances under various conditions (−AD, no AD; −PD, no PD). The average time required for a PO to move from the tip to sub-apical regions is indicated. Data points are provided as mean±s.e.m., *n*=2,000 simulations. AD is required for rapid mobility over short distances. It is noteworthy that curve for control is covered by ‘no PD' (−PD) and therefore not visible. (**d**) Diagrams show projections of POs, mixed in a cylinder of 10 μm × 2 μm in diameter under various conditions, captured after 250 s. It is noteworthy that simulations started with 15 blue and 15 red POs, placed at either end of the field (see Supplementary Movie 7). Scale bar, 2 μm.

We exploited our mathematical model to identify the relative contribution of (i) EE-driven enhanced AD, (ii) motor-driven DT along MTs (DT) and (iii) the actin-based PD to the growing tip, to the spatial organisation and mixing of POs (for parameters used in this modelling approach see Supplementary Table 3). The model predicts an even distribution of organelles (Fig. 6a, ‘Control'), whereas a ‘block' in AD and DT led to an increase of POs at the hyphal tip (Fig. 6a, ‘–DT/–AD'). When PD is removed, even distribution is restored (Fig. 6a, ‘–DT/–AD/–PD'). This is consistent with the idea that AD and DT oppose pole-ward actin-based forces. No PO clustering is predicted in the absence of PD alone (Fig. 6a, ‘–PD'), suggesting that AD and bidirectional PO transport are balanced. Finally, we examined the individual importance of DT and AD. Our model predicts that PO distribution would not be significantly affected in the absence of AD (Fig. 6a, ‘−AD'), but with moderate apical clustering of POs when DT is excluded (Fig. 6a, ‘−DT'). Thus, according to the model, motor-based transport is more important for distributing POs than AD. However, when both processes are absent, severe PO clustering is predicted to occur (Fig. 6a, compare ‘−DT/−AD' with ‘−DT'), indicating the involvement of both processes as being essential for even organelle distribution.

Even distribution of POs and LDs allows constant interaction between these and other organelles, required to perform their various cellular functions^2^,^3^,^4^. We observed interaction between POs in *U. maydis*, with transient connections formed between them that may serve to exchange lipids or metabolites^2^,^3^,^4^ (Supplementary Movie 7). Thus, both long- and short-range movements are of probable importance for the cell. We therefore used our model to test whether AD or DT is required for short-range and long-range mobility of POs. We positioned POs at the hyphal tip and simulated how long it takes for them to arrive at various distances behind the tip (first arrival time, FAT). We repeated these simulations 2,000 times, under control conditions, and after excluding PD. We found that the average FAT required to travel 25 μm from the tip is ∼0.7 h in both scenarios (FAT_25μm, Control_=0.662±0.013 h, *n*=2,000 simulations; FAT_25μm, −PD_=0.667±0.013 h, *n*=2,000 simulations; Fig. 6b, c; curves overlay each other in graphs; all time values in this experiment are mean±s.e.m. provided). This suggests that PD does not impair PO movement, when MT-associated processes are operational. In the absence of AD, the FAT increases slightly (FAT_25μm, −AD_=0.751±0.015 h, *n*=2,000 simulations; mean±s.e.m.). However, AD became important for movement over shorter distances, as FAT_3μm_ increases twofold when AD was excluded from the simulations (FAT_3μm, −AD_=0.096±0.002 h, *n*=2,000 simulations; FAT_3μm_, control=0.053±0.001 h, *n*=2,000 simulations; mean±s.e.m.; Fig. 6c). Conversely, DT is essential for long-range mobility of POs and its absence cause a 12-fold reduction in arrival time (FAT_25μm, −DT_=8.294±0.213 h, *n*=1,000 simulations; mean±s.e.m.; Fig. 6b). However, when both AD and DT are removed from the model, FAT increases by ∼200-fold (T_25μm, −DT/−AD_=129.017±11.874 h, *n*=100 simulations; Fig. 6b; mean±s.e.m.). This substantial increase is largely due to PD, because removing it reduces the FAT significantly (T_25μm, −DT/−AD/−PD_=28.35±2.42 h, *n*=100 simulation; mean±s.e.m.; Fig. 6b). This again highlights the importance of combinatorial activity of AD and DT for PO motility.

When considered together, the predicted variations in PO mobility from mathematical modelling suggest that AD and DT contribute to mixing of the peroxisomal compartment. To test this idea further, we modified our model and simulated the motion of two PO populations in a finite cylindrical space (10 μm in length × 2 μm in diameter), where PD is not taken into account. Consistent with the outcome of our motility simulations (Fig. 6b), POs show reduced mixing when DT was excluded, whereas the absence of AD has almost no effect (Fig. 6d and Supplementary Movie 8). However, PO mixing is dramatically reduced in the absence of AD and DT (Fig. 6d and Supplementary Movie 8). This is consistent with the synergy between the two processes in PO distribution and mobility.

In summary, our simulations suggest that (i) polar actin-based slow drift of POs provides the force for the clustering of POs at the growing tip, (ii) DT and, to a lesser degree, AD contribute to overcome these PD forces, to ensure even distribution, and (iii) AD and DT support PO mobility over short and long distances, respectively, and (iv) mobility and mixing of POs depends largely on both processes.

### MTs oppose F-actin to distribute POs in COS-7 cells

**Figure 7:**
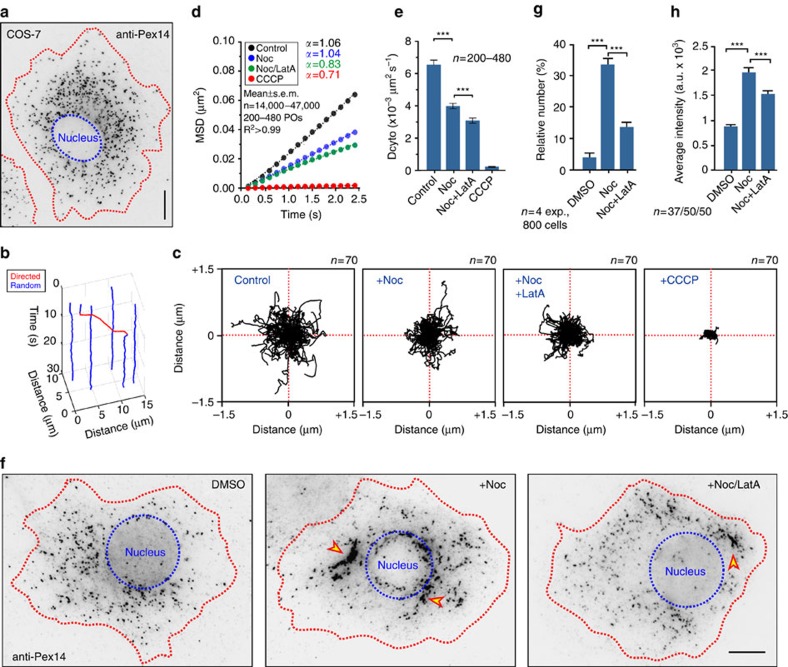
MT- and F-actin-based forces distribute POs in COS-7 cells. (**a**) Contrast inverted image of POs in a COS-7 cell, visualized by an anti-Pex14 antibody. Red line, cell edge; blue line, nucleus. Scale bar, 10 μm. (**b**) Switching between random (blue line) and directed motility (red line) of a PO in COS-7 cells. See Supplementary Movie 9. (**c**) Random motion of POs, labelled with GFP-SKL, in COS-7 cells treated for 30 min with DMSO (Control), nocodazole (+Noc) and nocodazole+latrunculin A (+Noc, +LatA). Plots summarize movement of 70 POs over 10 s. See Supplementary Movie 10. (**d**) MSD analysis of random PO motions in the presence of DMSO, nocodazole (Noc) or nocodazole+latrunculin A (Noc/LatA) and CCCP. All curves show linear increase (*α*∼1), confirming that the POs undergo diffusive movements. Each curve is based on analysis of 200–480 POs. (**e**) Diffusion coefficients (*D*_PO_) in the presence of DMSO (Control), nocodazole (Noc), a combination of nocodazole and latrunculin A (Noc+LatA) or CCCP. *D*_PO_ values were derived from MSD analysis (see above, Fig. 7d). Mean±s.e.m. is shown, *n*=200–480 POs. ***Statistical significance at *P*<0.0001, unpaired Student's *t*-test with Welch's correction. (**f**) Contrast inverted image of POs in a COS-7 cell, treated with DMSO, nocodazole (Noc) or nocodazole+latrunculin A (Noc+LatA) for 6 h. Depolymerization of MTs results in clustering of POs near the nucleus (arrowhead, +Noc), which is reduced when F-actin is also disrupted (arrowhead, +Noc/LatA). Red line, cell edge; blue line, nucleus. Scale bar, 10 μm. (**g**) Number of COS-7 cells with PO clusters after 6 h treatment with DMSO, nocodazole (Noc) or nocodazole+latrunculin A (Noc+LatA) for 6 h. Mean±s.e.m. is shown, *n*=4 experiments, 800 cells. ***Statistical significance at *P*<0.0001, unpaired Student's *t*-test with Welch's correction. (**h**) Intensity of anti-Pex14-Alexa488 antibody fluorescence in PO clusters in the presence of DMSO, nocodazole (Noc) or nocodazole+latrunculin A (Noc+LatA) for 6 h. Disassembly of MT and F-actin causes less PO clustering, indicated by lower fluorescence, than disrupting MTs alone. Mean±s.e.m. is shown, *n*=37–50 clusters. ***Statistical significance at *P*<0.0001, unpaired Student's *t*-test with Welch's correction.

To test the generality of the principles predicted by our mathematical model and the observation made in hyphae, we investigated PO positioning in mammalian cells, using COS-7 cells that contain GFP-SKL-labelled POs^41^. The organelles were evenly distributed around the nucleus, but largely excluded from the cell periphery (Fig. 7a). As in *U. maydis*, a small portion of the POs showed DT at a given moment in time (2.40±1.66%; *n*=90, 18 cells, 3 experiments, 8035 POs; mean±s.d.). It was shown that DT in COS-7 cells is based on MTs^42^. POs switched between random motions and directed motility (Fig. 7b and Supplementary Movie 9), again behaving as fungal POs. Random motions show diffusion-like properties (Fig. 7c,d, *α*=1.06±0.1; mean±s.e.m.) and were radically reduced when CCCP was added (Fig. 7c,d and Supplementary Movie 10), confirming previous reports in CHO cells^7^. Thus, mammalian and fungal PO diffusion is based on ATP-dependent biological activity. We next tested for a role of the cytoskeleton in random motion of mammalian POs by depolymerizing MTs with the inhibitor nocodazole and found that PO diffusion was also impaired (Fig. 7c,d, +Noc). PO motion was further reduced when F-actin was also disassembled by latrunculinA (Fig. 7c,d). A comparison of the estimated *D*_PO_ revealed that MTs have a greater impact on PO diffusion than F-actin (Fig. 7e). The results are consistent with our observations in *U. maydis*, suggesting that the cytoskeleton supports AD of POs in COS-7 cells.

Finally, we tested whether the disassembly of the cytoskeleton affects PO distribution. In the absence of MTs, PO clustered near the cell centre in ∼35% of all nocodazole-treated COS-7 cells (Fig. 7f,g, arrowhead; treatment for 6 h). This result confirms previous reports in mammalian cells^6^ and reflects our findings in *U. maydis* that MTs oppose intrinsic forces to enable even PO distribution. We next tested whether F-actin is involved in PO clustering. Interestingly, when both MTs and F-actin were disrupted simultaneously, significantly fewer PO clusters were found (Fig. 7f, +Noc/LatA and Fig. 7g; *P*<0.0001, unpaired Student's *t*-test with Welch's correction). These clusters also contained fewer POs, as indicated by significantly reduced fluorescence intensity after immuno-labelling of the PO protein Pex14 (Fig. 7h, *P*<0.0001, unpaired Student's *t*-test with Welch's correction). We conclude that MT-associated processes disperse POs, whereas F-actin-related activity induces PO clustering. This, as well as the motility behaviour of POs, resonates with observations in *U. maydis* and the predictions of our mathematical model. Thus, the fundamental principles underlying spatial organization of POs in the cytoplasm may be conserved from fungi to mammals.

## Discussion

In mammals and filamentous fungi, POs and LDs are evenly distributed in the cell, where they undergo short-range random motions. This even distribution and local random motion probably enables frequent organelle–organelle interaction, known to support their various cellular functions^2^,^43^,^44^,^45^. In this report, we provide evidence that even distribution of POs and LDs is actually an emergent consequence of these opposing cytoskeletal forces. We demonstrate that MT-associated EE motility is required to distribute organelles. In the absence of MTs or EE motility, POs and LDs cluster at the expanding hyphal tip, as is consistent with our previous results^18^. Similar PO aggregation was described in EE motility-defective mutants in *A. nidulans*^16^,^19^ and in a dynamin mutant in *P. chrysogenum*, and it was suggested that POs accumulate due to their apical formation^17^ and a defect in retrograde transport. In *U. maydis* and *A. nidulans*, MT plus ends are concentrated at the tip^24^,^46^. Consequently, minus-end-directed dynein motors are expected to remove newly formed POs from the tip. Indeed, apical PO clustering has been reported in dynein mutants in *A. nidulans*^16^. However, our data and work in *A. nidulans* show that deletion of plus-end directed kinesin-3 also causes polar PO clustering^16^,^18^. Here we provide an explanation for these seemingly conflicting observations. We show that existing POs and LDs move towards the tip of the hyphal cell, when MTs are disrupted, and that this PD is F-actin and myosin-5 based. However, what is the reason for this PD? To understand this, we need to consider that fungal hyphae are polar structures that extend by tip growth^25^. Such polar expansion is driven by the constant delivery of secretory vesicles to the apex^47^,^48^. We know little about the mechanism that underpins this vesicle delivery. However, a central role of myosin-5 in fungal polarized growth has been shown in several fungi^25^,^26^, including *U. maydis*^28^,^29^. One may argue that myosin-5 directly transports POs to the tip. However, we estimate that PD occurs ∼3,000-fold more slowly than the velocity we measured here for myosin-5 transport. Moreover, GFP_3_-Myo5 and POs do not co-localize. We therefore propose that F-actin-based trafficking of secretory vesicles towards the tip provides a perpetual cytoplasmic flux. Alternatively, very short-lived interactions of myosin-5 and POs, not time resolvable in our experiments, could cause the slow pole-ward motion.

Finally, we should consider whether polymerization of MTs or F-actin could generate the force for apical drift of POs. In *U. maydis*, MTs polymerize in anti-polar bundles towards both cell poles^23^. Although MT dynamics including polymerization and lateral bending of MTs could increase AD of POs, the bidirectional organization of the MT array makes a role in PD of the organelles unlikely. On the other hand, ∼90% of myosin-5 movements are directed to the cell tip, indicating that the actin array is unipolar, with plus ends directed to the hyphal tip. Therefore, actin treadmilling will create a retrograde flow, expected to oppose plus-end myosin motors, a scenario that was described for protein localization in mammalian stereocilia^49^. Consequently, cytoskeletal dynamics is not expected to cause the PD of POs. Taken together, we consider it most probable that pole-ward motility of myosin-5 generates the drift of POs that, when unopposed by MT-associated motility, moves POs and LDs towards the growing tip.

Diffusion in the cytoplasm is enhanced by the activity of molecular motors^12^,^14^,^15^. Organelles display diffusion-like random motion, which was suggested to be a consequence of Brownian thermal motion and, more importantly, random ATP-dependent ‘fluctuating forces' (overview in ref. 13). Our MSD analysis confirms that random motion of POs and LDs in *U. maydis* and COS-7 cells has diffusion-like properties (*α*∼1). Consistent with an important role of active processes, PO diffusive random motion is largely abolished when ATP levels are reduced. This is consistent with the idea that enzymatic activity, rather than thermal Brownian motion, underlies organelle random motion in both cell types. We also demonstrated that AD of POs in *U. maydis* depends largely on MTs. These cytoskeletal fibres enable constant bidirectional motility of EEs^50^, suggesting that endosome trafficking could provide the force that enhances PO random motion. Indeed, random PO motions are reduced drastically when EE transport is inhibited in *hok1* mutants. In fact, no significant difference in diffusion rates is found in the absence of MTs or when EE motility is blocked (*P*=0.9718). Thus, bidirectional EE motility along MTs is most likely to be the major force enhancing PO diffusive motion. This is reminiscent of membrane trafficking in plants, which drives hydrodynamic flow in the cytoplasm, thereby transporting molecules, small particles and, indirectly, moving other organelles^51^,^52^. Similarly, vesicle transport along MTs mediates cytoplasmic streaming in *Drosophila* oogenesis^53^ and myosin-5-based vesicle trafficking exerts force on nuclei in mouse oocytes^15^. Thus, a role for membrane trafficking in mixing of the cytoplasm and embedded organelles is apparently conserved across the Kingdoms.

We report that ∼95% of all POs show AD, whereas <5% of POs in *U. maydis* and COS-7 cells undergo motor-driven DT along MTs. Although this latter number is small, our mathematical modelling suggests that this DT is important for PO distribution, as well as PO mobility and mixing over long distances. This confirms previous modelling results, predicting significant increases in organelle–organelle interactions through DT^54^. At first glance, AD appears to be irrelevant for long-distance movement, PO mixing and establishment of an even distribution of POs in the hyphal cell. In fact, one could argue that AD is an irrelevant byproduct of constant EE motility, required to distribute the protein translation machinery^55^ and various organelles^18^. However, this is clearly not the case. First, our modelling predicts that AD increases the movement of POs over short distances (twofold increase of mean FAT to 3 μm from tip; Fig. 6c). POs interact with each other and increasing their mobility by AD supports this organelle–organelle communication. Second, AD adds robustness to long-distance PO mobility, PO distribution and mixing. Our simulations demonstrate that simultaneous removal of DT and AD dramatically affects these parameters. This is best illustrated by the effect on long-distance retrograde mobility, shown as FAT over 25 μm, which was decreased 12-fold when DT was ignored, but decreased ∼200-fold when AD was removed as well. However, why is AD on its own of little importance for PO mobility? To understand this, we have to consider that DT is both dominant and bidirectional; it, alone, is almost enough to mix and distribute POs in the cell. However, when DT is not operational, AD is the only process opposing PD. In the absence of both AD and DT, pole-ward F-actin-based membrane trafficking induces polar organelle drift and dramatically alters organelle distribution and retrograde PO diffusion.

**Figure 8:**
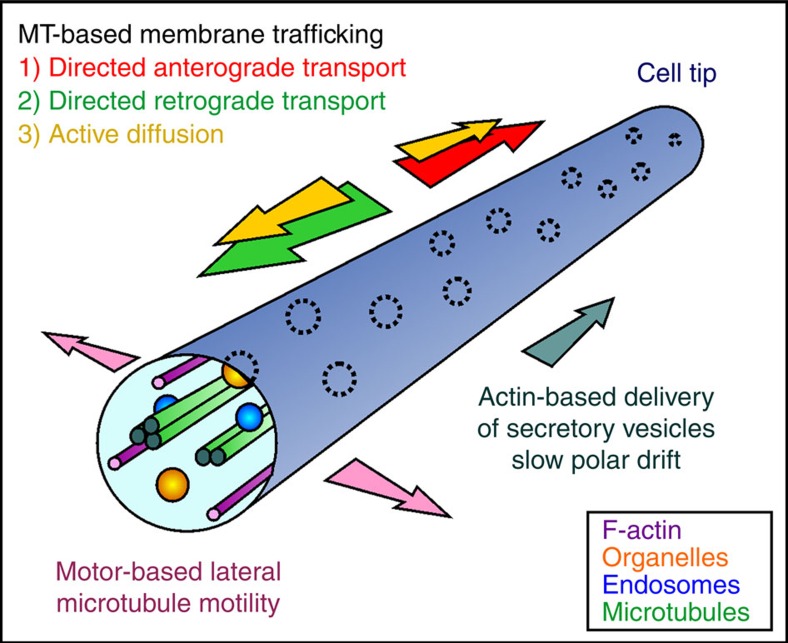
Even PO distribution in a hyphal cell is an emergent consequence of opposing cytoskeletal forces. MTs mix and distribute POs by supporting bidirectional EE motility that mediates anterograde (red arrow) and retrograde (green arrow) DT of POs, but also underlie their enhanced AD along the axis of the cell (yellow arrows). MT bending exerts lateral force (purple arrow) and increases the contact between POs and EEs. MT-based mechanisms oppose a slow pole-ward drift of POs (dark green arrow), which is mediated by myosin-5 at the cell periphery. Thermal diffusion plays only a minor role in PO mobility and is excluded from the diagram.

We have shown that biological activity is needed to evenly distribute and mix organelles in the cytoplasm. At first glance, this finding is surprising. Fick's law of diffusion postulates that thermal random motion will reduce gradients and thus, on average, should evenly distribute organelles in a cell. However, within the crowded cytoplasm, thermal Brownian motion of membranous organelles is restricted. This limitation in mobility is overcome by motor-dependent activity, which enhances random movement of organelles, a process named AD^14^,^15^. In *U. maydis*, constant bidirectional EE transport supports AD of POs and most probably LDs, suggesting that this process increases mobility of organelles. Indeed, modelling confirms this notion over short distances. However, the model also predicts that AD alone is not sufficient to mix POs or to ensure their even distribution in the cell. In fact, even distribution requires the cooperation of both AD and rare directed motility. Moreover, MTs need to explore the lateral space of the cell by motor-driven bending. We report here that all these motor-driven processes compensate for the tip-directed F-actin-based flux (Fig. 8). This pole-ward force is a result of continuous delivery of growth supplies to the expanding hyphal tip. As apical tip extension is a hallmark of filamentous fungi, such polar forces are an inevitable emergent phenomenon of polarized invasive growth. Finally, in COS-7 cells, POs aggregate when MTs are absent and such clustering is reduced when F-actin is also disrupted. Thus, myosin-related forces may act on mammalian POs and are opposed by MT-dependent transport processes. Alternatively, retrograde treadmilling in the peripheral actin network could account for PO clustering in COS-7 cells. Although more mechanistic insight remains elusive, our results highlight that the fundamental principles underpinning organelle positioning are common to fungal and mammalian cells.

## Methods

### Growth conditions

*U. maydis*. Liquid cultures were grown at 28 °C, shaking at 200 r.p.m., for 8–12 h in complete medium, supplemented with 1% (w/v) glucose^56^. Hyphal growth was induced by transferring the yeast-like cells to nitrate minimal medium, supplemented with 1% glucose (NM_Glu_), following published procedures^55^. Microscopic observation was started following additional 8–14 h growth at 28 °C, 200 r.p.m. in NM_Glu_. Temperature-sensitive mutant strain AB33Kin3^ts^_paGSKL or AB33Kin3^ts^GSKL was grown in CM_Glu_ for 8–12 h. Hyphal growth was induced in NM_Glu_ at permissive temperature (22 °C) for 8–14 h. To inactivate Kin3^ts^, these liquid cultures were transferred to restrictive temperature (32 °C), and PO distribution and random PO motion were monitored in permissive and restrictive temperature after 3 h or 30 min, respectively. The control strain AB33GSKL was treated accordingly.

*Mammalian COS-7 cells*. COS-7 (ECACC 87021302) and COS-7-GFP-SKL cells, which stably express GFP-SKL fusion protein^41^, were maintained in DMEM medium high glucose (4.5 g l^−1^) (Life Technology, Paisley, UK) supplemented with 10% FBS (w/v; Life Technology), 100 U ml^−1^ penicillin and 100 μg ml^−1^ streptomycin (Life Technology), at 37 °C in a 5% CO_2_-humidified incubator (Thermo Fisher Scientific, Waltham,USA).

### *U. maydis* strains and plasmids

Strains AB33paGFP_2_, AB33GSKL, AB33ΔKin3GSKL, AB33ΔHok1GSKL, AB33G_3_MyoV, AB33LifeactG and AB33GT were described previously^18^,^23^,^28^,^55^. Strain AB33GTub1GSKL allowed co-visualization of POs and MTs, and was generated by digestion of poGSKL^H^ (ref. 18) with BglI and integrated ectopically into AB33GT (ref. 23). The temperature-sensitive strain AB33ΔKin3Kin3^ts^_paGSKL was used to study inactivation of a mutant allele of kinesin-3 on PO distribution. It was obtained by digestion of plasmid pKin3^ts^ (ref. 23) with BsrGI, followed by ectopic integration into a *kin3*-null mutant strain AB33ΔKin3Kin3^ts^ (ref. 55). Successful integration was confirmed by studying the rescue of the morphological phenotype of AB33ΔKin3Kin3^ts^ at permissive (22 °C) and restrictive (32 °C) temperatures. This was followed by ectopic integration of plasmid popaGFP-SKL^C^, digested with EcoRV. Plasmid ppaGSKL^C^ was generated by fusing the PO targeting signal 1 (SKL=serine–lysine–leucine) to the carboxy terminus of paGFP (ref. 57), using primers GD112 and GD113 (Supplementary Table 4). The carboxin resistance cassette was amplified from paGRab5a, using primers GD110 and GD111 (Supplementary Table 4). Strain AB33G_3_Myo5GSKL was generated by ectopic integration of plasmid po^C^mChSKL (ref. 18), which was digested with AgeI, into strain AB33G_3_Myo5 (ref. 28). To generate strain AB33ΔRab5aGSKL, the plasmid po^C^GSKL (ref. 37) was digested with AgeI and ectopic integrated into strain AB33ΔRab5a (ref. 36). All fragments were ligated by yeast recombination^58^, using *S. cerevisiae* strain DS94 (MATα *ura3-52 trp1-1 leu2-3 his3-111 lys2-801*) and confirmed by restriction enzyme digestion. Strain AB33ΔMyo5_GSKL allowed the observation of POs in the absence of myosin-5. It was generated by digestion of poGSKL^C^ (ref. 18) with AgeI, followed by ectopic integration into AB33ΔMyoV (ref. 59). For the genotype of all strains, see Supplementary Table 5. For experimental usage of all strains, see Supplementary Table 6. *U. maydis* transformations were performed following published protocols^60^. In brief, protoplasts from 50 ml cell suspensions, grown overnight in YEPS medium (1% (w/v) yeast extract, 25 (w/v) bacto-peptone and 2% (w/v) sucrose) were generated by incubating with 7 mg ml^−1^ Novozyme 234 (NovoNordisk, Denmark) in SCS (20 mM sodium citrate pH 5.81 and 1 M sorbitol) for ∼15 min at room temperature. Cells were harvested by low-speed centrifugation, washed three times with cold SCS and resuspended in 500 μl cold STC (10 mM Tris-HCI pH 7.51, 100 mM CaCI_2_ and 1 M sorbitol). Fifty microlitres of this protoplast suspension were incubated with DNA and 1 μl heparin (stock: 15 mg ml^−1^ in water) for 30 min on ice. Five hundred microlitres STC/40% polyethylene glycol was added, followed by 15 min incubation on ice. The cell suspension was transferred onto regeneration-agar plates (1.0% (w/v) yeast extract, 2.0% (w/v) bacto-peptone, 2.0% (w/v) sucrose, 18.22% (w/v), sorbitol and 1.5% (w/v) agar) and incubated for 2 days at 28 °C before further analyses.

### Live-cell imaging

*U. maydis* microscopy was performed as previously described^30^. In brief, cells were placed on a 2% agarose cushion and observed using a motorized inverted microscope (IX81; Olympus, Hamburg, Germany), using a Plan-Apochromat × 100/1.45 numerical aperture total internal reflection fluorescence oil objective or UPlan-SApochromat × 60/1.35 numerical aperture oil objective (Olympus). Fluorescent proteins were excited by 70 mW solid-state lasers, at 488 and 561 nm, controlled by a VS-LMS4 Laser Merge System (Visitron Systems, Munich, Germany). Photobleaching experiments were performed using a 405-nm/60-mW diode laser, dimmed to 15 mW output power, which was controlled by a UGA-40 unit (Rapp OptoElectronic, Hamburg, Germany) and VisiFRAP-2D FRAP control software for Meta Series 7.5.x (Molecular Devices, Downingtown, PA). Simultaneous observation of mCherry and enhanced GFP fluorescence was performed using a Dual-View Micro Imager (Photometrics/Roper Scientific, Ottobrunn, Germany), equipped with a dual-line beam splitter (z491/561; Chroma Technology Corp., Olching, Germany), an emission beam splitter (565 DCXR; Chroma Technology Corp.), an ET-Band pass 525/50 (Chroma Technology Corp.) and a single band-pass filter (BrightLine HC 617/73; Semrock, Rochester, USA). Images were acquired using a cooled charge-coupled device camera (CoolSNAP HQ2; Photometrics/Roper Scientific). For temperature-dependent experiments, the objective lenses were cooled or heated using a metal hull connected to a water bath (Huber, Offenburg, Germany). The microscopic system control, all image processing and quantitative analysis was done using MetaMorph 7.5.x (Molecular Devices).

Mammalian COS-7 cells were observed using an Olympus IX81 microscope (Olympus Optical, Hamburg, Germany), equipped with a PlanApo × 100/1.40 oil objective and enhanced GFP filter sets (470/40 ET band-pass, beam-splitter T 495 LPXR and a 525/50 ET band-pass filter; Chroma Technology Corp.) and a TxRed HC filter set (562/40 BrightLine HC, HC beam-splitter BS 593 and a 624/40 BrightLine HC; Semrock). Cells were kept in a closed chamber in glass-bottom 35-mm petri dishes with 20 mm bottom well (Greiner Bio-One, Frickenhausen, Germany) in HEPES-buffered DMEM without phenol red (DMEM 1 ×, Gibco, Life Technology). Temperature was kept at 37 °C, using a temperature control system and a microscope objective heater (Visitron Systems). Image acquisition was performed as described above.

### Electron microscopy

For immuno-gold labelling, cells were aldehyde-fixed (0.5% glutaraldehyde in 0.2 M PIPES buffer pH 7.2), sedimented at 17,000 *g* and washed in fresh buffer followed by cryoprotection in 2.3 M sucrose in PBS (pH 7.2; 137 mM NaCl, 2.7 mM KCl, 8.1 mM Na_2_HPO_4_ and 1.5 mM KH_2_PO_4_). Eighty-nanometre cryosections, cut at −100 °C using a LN ultracryomicrotome (RMC Boeckeler Instruments Inc., Tucson, USA) were thawed and labelled with a rabbit polyclonal antibody against GFP (stock: 2 mg ml^−1^, diluted 1:200 in fish skin gelatin/PBS; GFP Tag Antibody A-11122, Life Technologies, Paisley, UK), followed by 10 nm Protein-A gold (BBI Solutions, Cardiff, UK). Subsequent contrasting was done with 2% (w/v) methylcellulose and 2% (w/v) uranyl acetate in water. Quantification of the labelling density was performed by taking 20 micrographs per sample with a JEOL JEM 1400 transmission electron microscope, operated at 120 kV and nominal magnification of 80k, equipped with a digital camera (Gatan ES1000W, Abingdon, Oxon, UK). Gold particles were counted within structures of interest and in the surrounding cytoplasm and related to area or membrane boundary length estimated by point or intersection counting, respectively. Gold particles were categorized as membrane-associated if the particle was located less than one particle width from a membrane profile. To quantify the size of POs and EEs, profiles of both organelles were sampled by systematically scanning the labelled sections and taking images at a nominal magnification of 150k. The mean diameter of organelles was estimated by averaging the measurements along the horizontal and vertical axis of the organelle profiles.

### Drug treatment

For disruption of MTs or F-actin cytoskeleton in *U. maydis*, 500 μl of the cell culture was supplemented with 30 μM benomyl (stock: 30 mM in dimethylsulphoxide (DMSO); Fluka, Sigma-Aldrich, Gillingham, UK) 20 μM latrunculinA (stock: 20 mM in DMSO, Life Technologies) or 100 μM of CCCP (Sigma-Aldrich) and incubated for 30 min in at 28 °C at 200 r.p.m. Control cells were incubated under identical conditions with 0.5 μl DMSO. Cells were placed on 2% (w/v) agarose cushions, supplemented with 30 μM benomyl, 20 μM latrunculin A or corresponding amounts of their solvent DMSO, respectively. Image series of 100 frames at 150 ms were acquired. The effective depolymerization of MTs or F-actin was tested in control experiments, using GFP–α-tubulin-expressing strain AB33GT and Lifeact-GFP-expressing strain AB33LifeactG. Reversibility of CCCP treatment was confirmed by washing cells, followed by 15–30 min incubation in fresh medium and monitoring the reappearance of PO movements.

To investigate the effect of cytoskeletal drugs or CCCP on mammalian PO motility, GFP-SKL-expressing COS-7 cells were grown in glass-bottom dishes (Greiner Bio-One). Nocodazole (10 μM; stock: 33.2 mM), 300 nM (long-term incubation) or 20 μM latrunculin A (short-term incubation; stock: 20 mM) or a combination of both, was added to the culture medium for 30 min or 6 h, followed by microscopic analysis in DMEM without phenol red (DMEM 1 ×, Gibco, Life Technology) at 37 °C for no longer than 1 h. To investigate the effect of cytoskeletal drugs on PO distribution, unlabelled COS-7 cells were grown on glass coverslips and were treated with 10 μM nocodazole, 300 nM latrunculin A, or both drugs simultaneously for 6 h, followed by fixation in 4% (v/v) paraformaldehyde in PBS pH 7.4. Cells were permeabilized with 0.2% (v/v) Triton X-100, blocked with 1% (w/v) BSA and incubated with rabbit polyclonal anti-Pex14 antibodies (kindly provided by D. Crane, Griffith University, Brisbane, Australia), followed by incubation with goat-anti-rabbit IgG conjugated to Alexa 488 (Life Technology). Effective disruption of MTs and F-actin was tested in parallel experiments, staining F-actin with Phalloidin-A594 (Life Technologies) and MTs with anti-α-tubulin (Sigma-Aldrich).

### Analysis of directed organelle and GFP_3_Myo5 motility

Directed motility and run length of POs were analysed in image series and in movies, taken from strain AB33GSKL. Directed motility was defined as rapid and continuous motility that lasted >2 μm. The percentage of motility was determined in a 15-s time interval. To analyse the percentage of POs or LDs, which showed DT at a given moment in time in *U maydis*, image series of 150 frames were acquired, using strains AB33GSKL or AB33Erg6G. Kymographs were generated and the total number as well as the number of motile POs/LDs were counted at three different time points (plain 10, 50 and 100). To analyse the percentage of POs, which showed DT at a given moment in time in COS-7 cells, 100 *z*-series with a *z*-distance of 500 nm and an exposure time of 100 ms were taken. From each *z*-series, a maximum projection was generated and out of those a movie was reconstructed. Owing to the size of the cells, two regions per cell, containing of up to 125 POs, were randomly chosen. The total number and the number of motile POs (within the 500-ms window) was counted at three different time points (plain 10, 50 and 80).To measure the flux of GFP_3_-Myo5, image series of 150 frames were acquired, using strain AB33G_3_Myo5, 150 ms exposure time and a 488-nm laser at 90% output power. Kymographs were generated from these image stacks using MetaMorph 7.5.x, and anterograde and retrograde flux was measured over 7 s at 5 μm behind the hyphal tip.

For co-localization of GFP_3_Myo5 and mCherry-SKL-containing POs, overnight-grown cells of strain AB33G_3_Myo5GSKL were shifted to hyphal growth as described above. For an accurate alignment of myosin-5 movements and POs, data acquisition was performed after calibration of the system, using 0.2 μm TetraSteck fluorescent microspheres (Thermo Fisher Scientific; diluted 1:10 in water). To this end, images of the beads in both channels were taken, using the dual imager (10% output power of 488 and 561 nm lasers, at 150 ms) and the channels were aligned using the ‘Split View' function in MetaMorph, using the defined parameters to align the acquired data. GFP_3_Myo5 motility events were detected in one image stack and the region of interest was transferred to the red-fluorescent image stack. Kymographs of both regions were generated and overlaid using MetaMorph. Data were compared using unpaired Student's *t*-testing, including Welch's correction to account for potential differences in variances, using Prism5.03 (GraphPad, La Jolla, USA).

### Estimation of motor numbers in moving GFP_3_Myo5 signals

The numbers of motors in distinct and moving GFP3Myo5 signals were estimated by comparison of fluorescent intensities to GFP-Nup107, a fluorescent nucleoporin. This followed previously published protocols, used to determine kinesin-3 and dynein motor numbers in *U. maydis*^30^,^61^. We assumed that myosin-5 motors in *U. maydis* contain two myosin heavy chains^62^, encoded by the *myo5* gene in *U. maydis*^29^. As a triple GFP gene was inserted in this native locus, all myosin-5 motors in the cells contain six GFP. To determine the number of motors in a moving GFP_3_Myo5 signal, the tip region of GFP_3_Myo5-expressing hyphae was photobleached and after 5 s short image series were captured. Moving signals were identified and their fluorescent signal intensity, corrected for the adjacent background, was determined in the first frame of the image sequence. This fluorescent intensity was compared with the intensity of individual fluorescent nuclear pores in strain FB2N107G, each containing 16 GFP-Nup107 molecule^30^,^61^. From this comparison, the number of myosin-5 motor complexes was estimated.

### Analysis of PO and LD distribution

To measure PO and LD distribution in hyphal cells of control, kinesin-3 and *hok1*-null mutants (strains AB33GSKL, AB33ΔKin3GSKL and AB33ΔHok1GSKL, AB33Erg6G and AB33ΔHok1mCRab5aErg6G), *z* axis image stacks were acquired with an exposure time of 150 ms and 200 nm steps in the *z*-direction. From these stacks, maximum projections were generated using MetaMorph 7.5.x and the average fluorescent intensity over the length of individual hyphal cells was measured using the line-scan function in MetaMorph 7.5.x. The measurements of each hyphal cell were transferred into the software Excel (Microsoft, Redmond, USA) and the mean intensity was calculated. Analysis of PO and LD distribution after disruption of the cytoskeleton in control strain AB33GSKL, AB33ΔMyo5GSKL and AB33Erg6G was done in an analogous way. Cells were treated for 5 h in 2–5 ml cultures with cytoskeleton inhibitors (30 μM benomyl, 20 μM latrunculin A or a combination of both) and were incubated at 28 °C, 200 r.p.m. Subsequently, PO distribution was measured as described above. The effect of inactivation of kinesin-3 on PO distribution was investigated in strain AB33Kin3^ts^_paGSKL. POs in hyphal cells were bulk photoactivated in a custom-built glass chamber, containing a coverslip, inside which the cell suspension was placed, and a liquid reservoir to prevent desiccation. Photoactivation was performed using a × 10 objective (Olympus) and a 405-nm laser at 100% output power. Successful photoactivation was confirmed by epi-fluorescent microscopy, using the 488-nm observation laser at 20% output power. Cells in the custom-built glass chamber were transferred into a 32 °C incubator for 3 h. *Z* axis image stacks were acquired at an exposure time of 150 ms and 200 nm steps in the *Z*-direction and analysed as described above.

### MSD and diffusion rate estimation

To analyse the random motions of POs, the MSD was calculated according to published methods^63^. Image series of GFP-SKL-expressing *U. maydis* and COS-7 cells were recorded at 150 ms interval and covering 15–45 s observation time. Random motions of POs were automatically detected using tracking software that determined the PO centre after Gaussian filtering (for details, see Supplementary Methods). Trajectory plots were drawn in MATLAB R2011b (MathWorks, Cambridge, UK), using trajectories that record 10 s of random motions. MSD calculations were done using MATLAB R2011b and curve fitting analysis was done in Prism 5.03. Diffusion coefficients *D*_PO_ were calculated from MSD of individual trajectories, using MATLAB R2011b or derived from fitted curves using Prism 5.03. Unpaired Student's *t*-testing, including Welch's correction to account for potential differences in variances, was done in Prism5.03. To calculate that axial and radial diffusion rates, the axis of *U. maydis* hyphal cells was determined using the image moments^64^ of the live-cell imaging data after applying a median threshold. Subsequently, individual trajectories were oriented according to this spatial information and *D*_PO_ for random movements along the axis (axial) and 90° to the axis (radial) was calculated as described. Nonlinear curve regression and F-tests for *D*_PO_ comparison were performed using the software GraphPad Prism 5.03. Further details of MSD analysis and *D*_PO_ estimation can be found in the Supplementary Methods.

### Mathematical modelling

The mathematical model of PO distribution along the cell was based on the findings that POs are distributed by (i) anterograde-directed motility, (ii) retrograde-directed motility, (ii) AD implemented by MT-based membrane trafficking and (iv) F-actin-based PD. These factors were combined in a three-population modelling approach^65^. Our model assumes that three populations of POs contribute to the entire distribution pattern of POs in the cell. The first two populations represent POs that bind to EEs and undergo anterograde or retrograde movements along MTs; switching between these two populations is determined by the turning of PO motility. The remaining population represents the POs that undergo AD and slow pole-ward drift. POs can switch between random motion and DT, thereby allowing transition between all three populations. The directed motility to random movement switching rate was determined from experimentally measured PO run lengths. The transition rate from random to directed motility was obtained from image sequences. To this end, POs were observed at 4.5 s intervals and the number of POs that switched from random to DT counted. The switching rate was determined by fitting the proportion of switching events to a one phase association function. Taking into account that MT bending enhances lateral interaction of POs with moving EEs, axial *D*_PO_s and switching rate to directed motility were estimated from randomly chosen POs. Model predictions of PO distributions were calculated numerically in the software Maple 17 (Maplesoft Europe Ltd, Cambridge, UK). The motility of individual POs and their FAT to a distance distal from the hyphal tip were simulated in Dev C++ (http://www.bloodshed.net/devcpp.html). Further details on the mathematical model and simulations can be found in the Supplementary Methods.

### Data availability

The data that support the findings of this study are available from the corresponding author on request.

## Additional information

**How to cite this article:** Lin, C. *et al*. Active diffusion and microtubule-based transport oppose myosin forces to position organelles in cells. *Nat. Commun.* 7:11814 doi: 10.1038/ncomms11814 (2016).

## Supplementary Material

Supplementary Information Supplementary Figures 1-5, Supplementary Tables 1-6, Supplementary Methods, Supplementary References

Supplementary Movie 1 PO Motility in U. maydis.

Supplementary Movie 2 Motility of POs along MT in U. maydis.

Supplementary Movie 3 Peripheral motility of GFP3-Myo5 in U. maydis.

Supplementary Movie 4 Random PO motility in the presence of inhibitors in U. maydis.

Supplementary Movie 5 Random motion of POs near MTs in U. maydis.

Supplementary Movie 6 MT bending and PO motility in U. maydis.

Supplementary Movie 7 Interaction of POs in U. maydis.

Supplementary Movie 8 Modelling of PO mixing.

Supplementary Movie 9 PO motility in COS-7 cells.

Supplementary Movie 10 Random PO motility in mammalian COS-7 cells.

## Figures and Tables

**Table 1:** Dimensions and numbers of cytoskeletal elements and organelles.

	Dimension	Reference
Hyphal diameter	2.03±0.03 μm (3; 58 cells)*	This study
PO diameter	237.01±8.68 nm (45)*	This study
EE diameter	187.35±11.4 nm (56)*	This study
LD diameter	∼300 nm	Ref. 66^66^
Number of actin cables	4.64±1.36 (11)*	This study
Diameter secretory vesicle	30–50 nm	Ref. 67^67^
Number of bundles	2	Ref. 23^23^
Number of MTs in bundle	3	Refs 23,38^23^,^38^
Diameter of a MT bundle	≈50 nm	Ref. 38^38^
Distance organelle to MT	17±2 nm/∼25 nm	Refs 68,69^68^,^69^
Diameter an actin filament	7–8 nm	Ref. 70^70^
Length of myosin	≈60 nm	Ref. 62^62^

EE, early endosome; LD, lipid droplet; MT, microtubule; PO, peroxisome.

^*^Mean±s.e.m. (sample size).
