# Supplementary material for: Active diffusion and microtubule-based transport oppose myosin forces to position organelles in cells
Source: Nat Commun. 2016 Jun 2;7:11814. doi: 10.1038/ncomms11814 (PMC4895713; doi:10.1038/ncomms11814)
Supplement: Supplementary Information — Supplementary Figures 1-5, Supplementary Tables 1-6, Supplementary Methods, Supplementary References [file ncomms11814-s1.pdf]

Supplementary Figures and Legends

Supplementary Figure 1

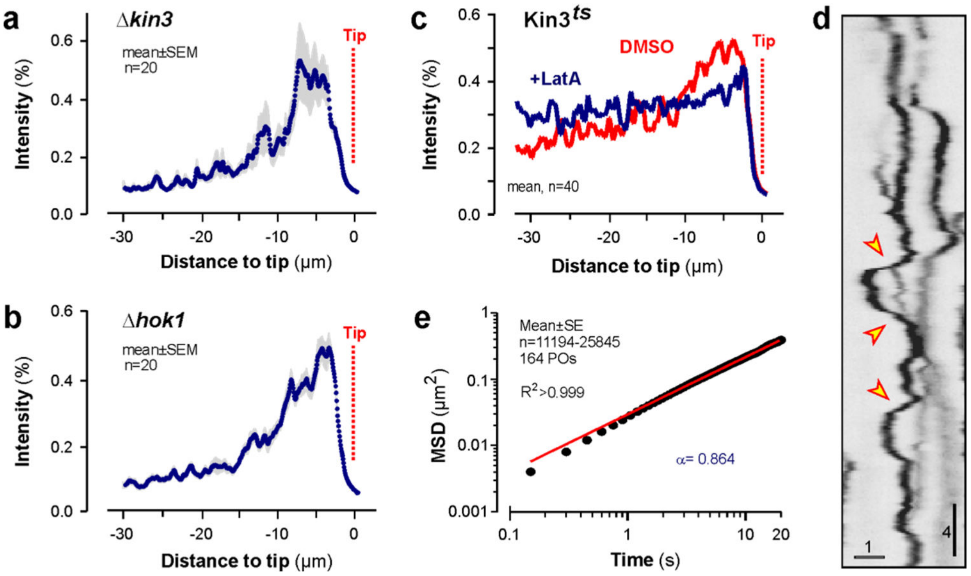

Supplementary Figure 1. PO distribution and motility in hyphal cells of *U. maydis*.

a, Average fluorescence intensity profile of GFP-SKL in kinesin-3 null mutants (Δ*kin3*). Each data point represents the mean ± SEM of measurements in 20 cells from two experiments. The position of the cell tip is indicated.

b, Average fluorescence intensity profile of GFP-SKL in *hok1* null mutants. Note that Hok1 is associated with EEs and required for their motility[1]. EEs are also mediating directed transport of POs along MTs (ref. 2). Consequently, the distribution curve is reflecting the absence of active diffusion and directed transport. The position of the cell tip is indicated. Each data point represents mean ± SEM of measurements in 20 cells from two experiments.

c, Average fluorescence intensity profile of GFP-SKL in temperature-sensitive $kin3^{ts}$ mutants after 2h of treatment with the solvent DMSO (red profile; and 20 mM

Latrunculin A (blue profile; +LatA). Each data point represents the mean ± SEM of measurements in 40 cells from two experiments. The position of the cell tip is indicated.

d, Contrast-inverted kymograph showing randomly moving POs (organelles labelled with GFP-SKL) in control cells. Scale bars, 4 s (vertical) and 1 µm (horizontal).
e, Graph showing MSD curve of random PO motility in untreated hyphal cells of *U. maydis*. The curve grows close to linear over 20 s (best fitting exponent α= 0.864, red line), indicating diffusion that is slightly restricted. Analysis is based on 164 trajectories.

Supplementary Figure S2

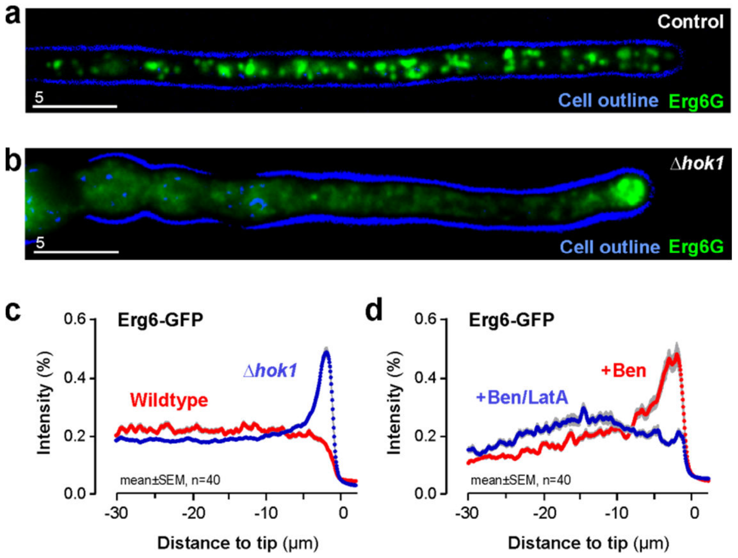

Supplementary Figure 2. Distribution of lipid droplets.

a, Lipid droplets, labelled with the putative methyltransferase Erg6-GFP (Ref. 2) in a hyphal cell. Note that LDs have a tendency to form subapical clusters, which was considered a methodological artefact and not included in the analysis. Scale bar, 5 µm.

b, Distribution of LDs in a *hok1* null mutant. Note that cells showed occasionally also subapical clustering. This was rare and excluded as considered artificial[2]. Scale bar, 5 µm.

c, Average intensity scans over the apical 30 µm in wild-type and a Δ*hok1* mutant cells. Each data point represents mean ± SEM, *n*=40 cells.

d, Average intensity scans over the apical 30 µm in wild-type cells treated with benomyl (+Ben) and simultaneously with benomyl and latrunculin A (+Ben/LatA). Each data point represents mean ± SEM, *n*=40 cells.

Supplementary Figure 3

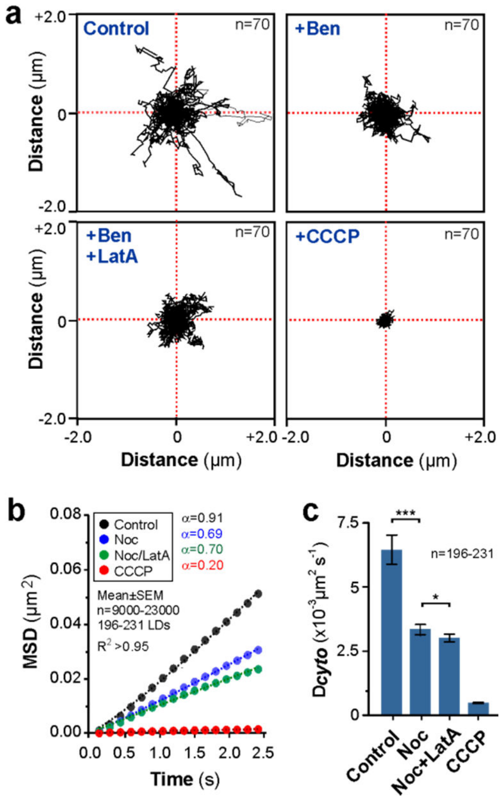

Supplementary Figure 3. Active diffusion of LDs.

a, Random motility of LDs in hyphal cells of *U. maydis*, treated for 30 min with DMSO (Control), benomyl (+Ben), a combination of benomyl and latrunculin A (+Ben, +LatA) or CCCP (+CCCP). Plots summarise 70 trajectories, starting at the centre and describing random motility of 70 LDs over 10 s. Almost no motility was found when ATP was depleted (+CCCP), suggesting that random PO motility is largely due to biological processes.

b, MSD analysis of oscillatory movements of LDs motility in the presence of the solvent DMSO (Control, black), benomyl (Ben, blue), a combination of benomyl and latrunculin A (Ben/LatA, green) or CCCP (red curve). Most curves show linear increase, with best fitting exponent α values around 1, confirming that the LDs undergo diffusion. Each curve is based on analysis of 193-231 LDs.

c, Diffusion coefficients of LDs in the presence of the solvent DMSO (Control), benomyl (Ben), a combination of benomyl and latrunculin A (Ben+LatA), or CCCP. Diffusion coefficients were derived from MSD analysis (see above, Supplementary Fig. 3b). Mean ± SEM is shown; bars based on 193-231 LDs. ***= statistical significance at $P<0.0001$, *= statistical significance at $P<0.05$, unpaired Student's T-test.

Supplementary Figure 4

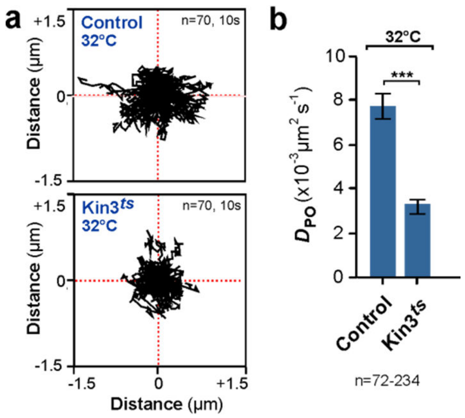

Supplementary Figure 4. Diffusive motion of POs in a temperature-sensitive kinesin-3 mutant.

a, Random motility of POs in control and conditional *kin3*^{ts} hyphal cells of *U. maydis* at restrictive (32°C) temperature. Plots summarise 70 trajectories, starting at the centre and describing random motility of 70 POs over 10 s.

b, Diffusion coefficients of in control and conditional *kin3*^{ts} hyphal cells of *U. maydis* at restrictive (32°C) temperature. Mean ± SEM is shown; bars based on 170-234 POs. ***= statistical significance at $P<0.0001$, *= statistical significance at $P=0.0034$, unpaired Student's T-test.

Supplementary Figure 5

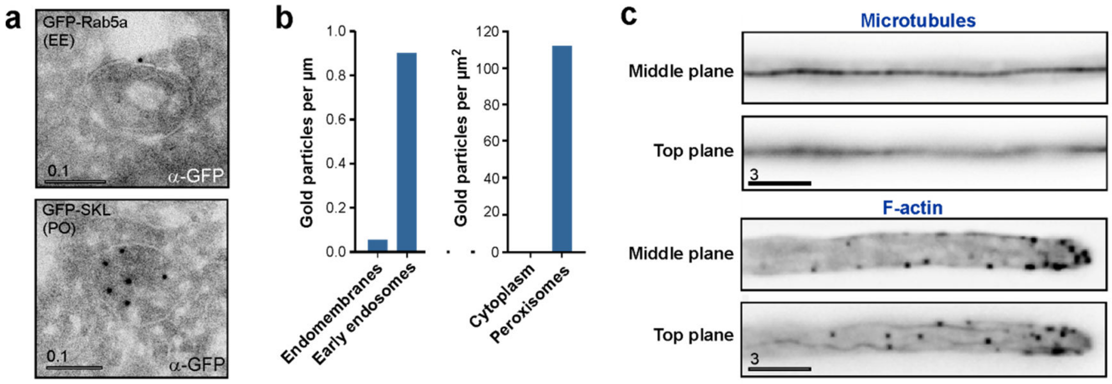

Supplementary Figure 5. Immuno-gold staining of organelles, model prediction of PO distribution and spatial organisation of the cytoskeleton.

a, Electron micrographs showing immuno-gold staining of GFP-Rab5a (upper panel) and GFP-SKL (lower panel). Scale bars, 0.1 µm.

b, Densities of GFP gold labelling estimated by point or intersection counting related to gold counts.

c, Spatial organisation of MTs and F-actin in hyphal cells of *U. maydis*. MTs are located centrally, while actin cables are concentrated at the cell periphery. Note that these peripheral F-actin cables allow streaming of Myo5 at underneath the cell surface (see Supplementary Movie 5). Scale bar, 3µm.

Supplementary Tables

Supplementary Table 1: The values for the axial diffusion rate and binding rate in the model for the wild type cell

Quantity	***Value***
D: axial diffusion rate (μm^2 s^{-1})	0.0137±0.0001
w_a: binding rate (s^{-1})	0.0034±0.00006

Supplementary Table 2: The measured and inferred rates in the model (S1-S3) for the wild type cell

Quantity	***Value***	***Estimate used***
v: net drift velocity in cytoplasm (μm s^{-1})	0.00044	See caption
M: run length (μm)	*6.5±0.61 (n=60)*	direct measurement
u: long-range transport velocity (μm s^{-1})	*1.9±0.05 (n=60)*	direct measurement
P_{turn}: Proportion of turning in directed transport (%)	*41*±1.45 (n=*279*)	direct measurement
w: turning rate (s^{-1})	0.12	$(uP_{turn})/M$
w_d: unbinding rate (s^{-1})	0.17	$u/M - w$

The net drift velocity v was estimated via fitting density profile from the model (S0) to experimental measured intensity for Δ*kin3* mutant cells.

Supplementary Table 3: The parameters used for hypothetical scenarios

Quantity	***<u>control</u>***	***<u>no actin drift</u>***	***<u>no active diffusion</u>***	***<u>no directed transport</u>***	***<u>no directed transport & no active diffusion</u>***	***<u>no directed transport & no active diffusion & no actin drift</u>***
D: (μm^2 s^{-1})	0.0137	√	0.0030	√	0.0030	0.0030
u: (μm s^{-1})	1.9	√	√	n/a	n/a	n/a
w_a: (s^{-1})	0.0034	√	√	0	0	√
w_d: (s^{-1})	0.17	√	√	∞	∞	∞
v: (μm s^{-1})	0.00044	0	√	√	√	0
w: (s^{-1})	0.12	√	√	n/a	n/a	n/a

‘ √ ’ : indicates the value from the control data and its specific values are in Supplementary Table 1 and Supplementary Table 2.

Supplementary Table 4. Primers used in this study

Primer	Sequence (5' to 3')
GD110	TAAGCTGTCAAACATGAGAATTCATCGATGGCGGCCGCACGGGGATCTTC
GD111	CTTAATTAAGGATCCGGCGCGCCGCGGCCGCACGCTAAGTGGAGTTGTCC
GD112	TATTTGAGAAGATGCGGCCAGCAAAACTAACTGAAGCTTGCATGCCTGCA
GD113	TGCAGCCGGGCGGCCGCTTTAAAGCTTCGACTTGTACAGCTCGTCCATGC

Supplementary Table 5. Strains and plasmids used in this study

Strain name	Genotype	Reference
AB33GSKL	*a*2 P*narb*W2 P*narb*E1, *ble*^{R}/po^{C}GSKL	Ref. 18
AB33ΔKin3Kin3^{ts}paGSKL	a2 P*narb*W2 P*narb*E1, *ble*^{R}, Δ*kin3, nat*^{R}/pKin3^{ts}/po^{C}paGSKL	This study
AB33GRab5a	*a2 PnarbW2 PnarbE1, ble*^{R} /poGRab5a	Ref. 61
AB33ΔKin3Kin3^{ts}GSKL	*a2* P*narb*W2 P*narb*E1, *ble*^{R}, Δ*kin3, nat*^{R}/pKin3^{ts}/po^{C}GSKL	This study
AB33LifeactG	*a2* P*narb*W2 P*narb*E1, *ble*^{R}/ po^{C}LifeactG	Ref. 28
AB33G$_3$Myo5	*a2* P*narb*W2 P*narb*E1, *ble*^{R}, P*myo5-3egfp-myo5, hyg*^{R}	Ref. 28
AB33ΔMyo5GSKL	*a*2 P*narb*W2 P*narb*E1, *ble*^{R} Δ*myo5, hyg*^{R}/po^{C}GSKL	This study
AB33G$_3$Myo5GSKL	*a*2 P*narb*W2 P*narb*E1, *ble*^{R}, P*myo5-3egfp-myo5, hyg*^{R}/po^{C}GSKL	This study
FB2N107	*a2b2* P*nup107-nup107-egfp*, *ble*^{R}	Ref. 30
AB33GTub1GSKL	*a*2 P*narb*W2 P*narb*E1, *ble*^{R}/poGTub1^{C}/po^{H}GSKL	This study
AB33ΔHok1GSKL	*a*2 P*narb*W2 P*narb*E1, *ble*^{R}, Δ*hok1, nat*^{R}/po^{C}GSKL	Ref. 18
AB33ΔRab5aGSKL	*a*2 P*narb*W2 P*narb*E1, *ble*^{R}, Δ*rab5, nat*^{R}/po^{C}GSKL	This study
AB33ΔKin3GSKL	*a*2 P*narb*W2 P*narb*E1, *ble*^{R}, Δ*kin3, nat*^{R}/po^{H}GSKL	Ref. 18
AB33nRFP	*a2 PnarbW2 PnarbE1, ble*^{R}/poNLS3RFP	Ref. 30
AB33GT	*a*2 P*narb*W2 P*narb*E1, *ble*^{R}/poGTub1^{C}	Ref. 23
AB33Erg6G	*a2* P*narb*W2 P*narb*E1,*ble*^{R} /poErg6G	Ref. 18
AB33ΔHok1mCRab5aErg6G	*a2* P*narb*W2 P*narb*E1, *ble*^{R}, Δ*hok1*, *nat*^{R}/pomChRab5a /poErg6G	This study
poGRab5a	P*otef-egfp-rab5a, nat*^{R}	Ref. 61
poNLS3RFP	P*otef-gal4s-mrfp-mrfp-mrfp, nat*^{R}	Ref. 30
po^{C}GSKL	P*otef-egfp-* SKL*, cbx*^{R}	Ref. 37
po^{H}GSKL	P*otef-egfp-* SKL*, hyg*^{R}	Ref. 18
pKin3^{ts}	P*kin3-kin3*^{ts}, *hyg*^{R}	Ref. 23
po^{C}paGSKL	P*otef-paegfp-SKL, cbx*^{R}	This study
po^{C}LifeActG	P*otef-LifeAct-egfp, cbx*^{R}	Ref. 28
poGTub1^{C}	P*otef-egfp-tub1, cbx*^{R}	Ref. 40
poErg6G	P*otef-erg6-egfp, cbx*^{R}	Ref. 18
pomChRab5a	P*otef-emcherry-rab5a, nat*^{R}	Ref. 61

a, *b*: mating type loci; P: promoter; -: fusion; Δ: deletion; *hyg*^{R}: hygromycin resistance; *ble*^{R}: phleomycin resistance; *nat*^{R}: nourseothricin resistance; *cbx*^{R}: carboxin resistance; *otef*: constitutive promoter; /: ectopically integrated; *E1*, *W2*: genes of the *b* mating type loci; *egfp*: enhanced green fluorescent protein; NLS, nuclear localization signal of the GAL-4 DNA binding domain from pC-ACT1 (Clontech); nup107, nucleoporin; *rab5a*, small endosomal Rab5-like GTPase *kin3*: kinesin-3; *hok1:* hook motor adapter; *Tub1*: α tubulin; SKL, peroxisomal targeting sequence; ^{ts}, temperature-sensitive allele; *pagfp,* photo-activatable GFP; LifeAct:: 17 amino acid peptide that binds F-actin; *erg6*, sterol-24-C-methyltransferase

Supplementary Table 6. Experimental usage of *U. maydis* strains

Strain name	Type of experiment	Figure or Movie
AB33nRFP	Overview of the hyphal cell	Figs. 1a
AB33_GSKL	Analysis of PO distribution and motility	Figs. 1b,c, 2a,b,d, 4a-h, 5c, Supplementary Fig. 1d, Supplementary Fig. 2a, 5a,b, Supplementary Movie 1, 4, 7
AB33ΔKin3_GSKL	Analysis PO distribution	Fig. 1b, Supplementary Fig. 1a
AB33ΔKin3_Kin3^{ts}_paGSKL	Analysis of PO pole-ward shifting	Fig. 1d,e
AB33GRab5a	Immuno-gold staining	Supplementary Fig. 5a,b
AB33LifeactG	Actin patches and cables distribution	Figs. 2c, Supplementary Fig. 5c
AB33G_3Myo5	GFP$_3$-Myo5 motility and number analysis	Fig. 3a,c, Supplementary Movie 3
AB33G_3Myo5GSKL	Co-observation myosin-5 and POs	Figs. 3f
FB2N107G	Analysis of myosin-5 numbers	Figs. 3b
AB33ΔMyo5_GSKL	Analysis of PO distribution	Figs. 3d,e
AB33ΔHok1_GSKL	Analysis of PO distribution and motility	Figs. 4g,h, 5c, Supplementary Fig.1b
AB33ΔRab5a_GSKL	Analysis of random PO motility	Figs. 4g
AB33GTub1_GSKL	Co-observation of POs and MTs	Figs. 5b, Supplementary Movie 2, 5, 6
AB33ΔKin3_Kin3^{ts}_GSKL	Analysis of PO distribution and motility	Supplementary Fig. 1c, 4a, 4b
AB33GT	Microtubules distribution	Supplementary Fig. 5c
AB33Erg6G	Lipid droplets distribution	Supplementary Fig. 2a,c,d, Fig. 3a,b,c
AB33ΔHok1_mChRab5a_Erg6G	Analysis of LD distribution	Supplementary Fig.3b,c

Supplementary Methods

Mean square displacement analysis and diffusion rate estimation

To analyse the spatial-temporal spreading of LDs and POs, the mean square displacement $MSD(t)\ = <|\boldsymbol{x}(t'+t)-\boldsymbol{x}(t')|^2>$ was calculated, where $|\cdots|$ represents Euclidean distance, $<\cdots>$ represents an ensemble average, and $\boldsymbol{x}(t) = (x(t), y(t))$ is the time-dependent trajectory position of the centre of an organelle. The local behaviour of a PO in Fig. 4b and Fig. 7b was classified by calculating the $MSD(t)$ using an ensemble average with a rolling window $(t-5s, t+$

$5s$) and fitting the $MSD(t)$ to a power law of the form At^{α} in the time interval $0 - 3s$. To classify the behaviour, for $\alpha > 1.6$ the motion was classified as directed, while for $\alpha < 1.6$ it was classified as diffusive. The $MSD(t)$ curves were calculated using an ensemble average over the entire trajectories of all tracked diffusive organelles and the exponents α were obtained by fitting the $MSD(t)$ to the form At^{α} in a time interval $0 - 2.5$ s (Fig. 4c, 7d, Supplementary Fig. 3b) and in a time interval $0 - 20$ s (Supplementary Fig. 1e),

The diffusion coefficients D were estimated by fitting $MSD(t)$ to a linear function $4Dt$ in a time interval $0 - 1.8s$ for each tracked diffusive-like PO (Fig. 4e, 4g) and LD (Supplementary Fig. 3c) in *Ustilago* hyphal cells and in a time interval $0 - 3s$ for each tracked diffusive PO in COS-7 cells (Fig. 7e). Mean and standard errors were calculated for the diffusion rates and student-t tests were performed to detect significant differences between experiments. For the axial and radial diffusion coefficients in Fig. 2d, as well as for the control experiments used in the mathematical modelling, the cell axis was determined through an automatic imaging process (described in the method part) and each trajectory $\boldsymbol{x}$(t) was rotated so that its first and second components correspond to axial and radial directions; the $MSD(t)$ in the axial (respectively radial) direction was calculated using an ensemble average of tracked diffusive POs for the first (respectively second) component of $\boldsymbol{x}$(t). These 1-dimensional $MSD(t)$ were fitted to a linear function $2Dt$ in a time interval $0 - 3s$ to get the best fit for the diffusion rate D. An F-test was performed for the significant difference between the best fitting diffusion coefficients in axial and radial direction (Fig. 2d). Nonlinear curve regression and F tests for diffusion coefficient comparison

were performed using the software Prism 5.03 (GraphPad Software, San Diego, USA).

Modelling the distribution of peroxisomes in mutant Δ*kin3*

The situation for the mutant *Δkin3* is simpler than the wild type – there appears to be no fast long-range directed motion of early endosomes. We took a simple drift diffusion model with no flux at the ends:

$$\frac{\partial\rho}{\partial t}=v\frac{\partial\rho}{\partial x}+D\frac{\partial^2\rho}{\partial x^2}\text{ for }x_0\leq x\leq x_L,\quad v\rho(x,t)+D\frac{\partial\rho(x,t)}{\partial x}=0\text{ at }x=x_0,x_L. \qquad \text{(S0)}$$

Here $x = x_0$ represents the cell tip, $x = x_L$ represents the other end of the region of interest and t represents time in seconds. Although the diffusion rate was estimated from the mean square displacement to be $D = 0.0049 \pm 0.00004um^2/s$, a reliable and direct measurement of the slow drift velocity v was not possible with the current time and spatial resolution. The model (S0) predicted a steady profile that is exponential of the form $\rho(x) = A\exp\left(\frac{-v}{D}x\right)$. We used this model to infer a value for this drift: we experimentally measured the average intensity profile in steady state in the $\Delta kin3$ mutant and fitted this intensity profile to $\rho(x) = A\exp\left(\frac{-v}{D}x\right) + B$ for $7 \leq x \leq 34\ um$ using nonlinear regression implemented in GraphPad Prism 5.03; parameter B concerns the background intensity in the experimental data. The best fit $\frac{v}{D} = 0.09 \pm 0.005\ um^{-1}$ was used to give $v\ = 0.00044\ um\ s^{-1}$.

Modelling the distribution of peroxisomes in wild type cells

In order to understand the coordination between the mechanisms that are transporting POs within the wild type cells, we construct an extension of the dynamical model (S0) for the distribution of POs along a portion of the cell $x_0 \leq x \leq x_L$. We postulate that POs move between three populations: $\rho_{1(2)}(x,t)$ is the density

of POs that are propagating actively and rapidly away from the tip (to the tip) carried by early endosomes (EEs, ref. 2), while $\rho_3(x,t)$ is the density of POs within the cytoplasm and that is undergoing both diffusion with rate D and a slow but deterministic drift with velocity v towards the tip. We assume that the propagation of the direct-transported POs is at velocity u in the respective direction and changes direction with a rate w; we assume POs in directed transport unbind from EEs at a rate w_d and those in cytoplasm bind to EEs and move in directly along MTs with a rate w_a. Thus the dynamics of the three populations is modelled by the following system of coupled partial differential equations:

$$\frac{\partial \rho_1}{\partial t} = -u\frac{\partial \rho_1}{\partial x} + \frac{w_a}{2}\rho_3 + w\rho_2 - (w_d + w)\rho_1 \quad \text{(S1)}$$

$$\frac{\partial \rho_2}{\partial t} = u\frac{\partial \rho_2}{\partial x} + \frac{w_a}{2}\rho_3 + w\rho_1 - (w_d + w)\rho_2 \quad \text{(S2)}$$

$$\frac{\partial \rho_3}{\partial t} = v\frac{\partial \rho_3}{\partial x} + D\frac{\partial^2 \rho_3}{\partial x^2} - w_a\rho_3 + w_d(\rho_1 + \rho_2) \quad \text{(S3)}$$

Persistence of directed transport could lead to accumulation at the ends[3,4]. However, in the context of POs in *Usilago* hyphal cells, the directed motion of POs is driven by EE motion and, consequently, the behaviour of POs at the ends reflects that of EEs. It has been shown in *Usilago* hyphal cells that EEs do not typically fall off the track[5], or form clusters at the cell tip[6]. Instead, they rapidly move away from the tip due to dynein activity. Therefore, at the ends of the domain, we assume that the directly-transported POs are recycled: a PO reaching the ends of the domain is assumed to immediately change direction and return. For the cytoplasmic POs we assume that there is no net flux at the ends of the domain. These correspond to applying boundary conditions on the fluxes at $x = x_0, x_L$

$J_1 = -J_2$ and $J_3 = 0$ (S4)

where the fluxes are $J_1 = u\rho_1, J_2 = -u\rho_2$ and $J_3 = -v\rho_3(x,t) - D\frac{\partial\rho_3(x,t)}{\partial x}$. Steady solutions for (S1-S3) can be found by superposition of eigenmodes of the form $\rho_i(x) = B_{ij}e^{\lambda_j x}$ where B_{ij}, λ_j $(i = 1,2,3;\ j = 1,2,3,4)$ are eigenvectors and eigenvalues for the corresponding linear problem. Note there is a uniform solution $\lambda_1 = 0$ but this does not satisfy (S4) except for the special case where there is no drift ($v = 0$). For typical parameters we can only satisfy (S4) for a specific combination of the eigenmodes.

We next estimated the parameters in (S1-S3) and used these and boundary conditions (S4) to predict the steady distribution of POs in the wild type. We note that measurements of the $MSD(t)$reveals more structure in the data if we align the cells to measure the axial and radial diffusion rates separately and if we restrict to certain populations of POs within the cell. However, the MT dynamics enhances lateral interaction of POs with moving EEs and this presumably has an effect both on axial diffusion and on the binding rate to moving EEs. We therefore estimated the axial diffusion coefficients and binding rate from randomly chosen POs (regardless of relative distance to the MT). To estimate the binding rate w_a, we counted at every 4.5 s the number of POs that have switched from random to directed transport as well as the total number of POs observed, and determined the binding rate by fitting the proportion of first binding events as a function of time duration, to a one phase association $1 - e^{-w_a t}$ with binding rate w_a. The diffusion rate was estimated from $MSD(t)$ using an ensemble average over randomly selected diffusive POs. Other parameters in the model (S1-S3) were estimated directly from experimental data and are shown in Supplementary Table 2. Using estimated parameters from Supplementary Table 1 and Supplementary Table 2, our model (S1-S4) predicted a

distribution (shown in Fig. 5c) which well agrees with the measured distribution of POs for the wild type cells. Numerical calculations of the predicted distributions from the model were performed in Maple 17 (Maplesoft Europe Ltd., Cambridge, UK). We remark here that over infinite time and infinite space, directed transport along MTs can be viewed as another "diffusion-like" process[7], which spreads POs at a rate described by $u^2/(2w)$=15 μm^{2}s^{-1} (using velocity and the turning rate, Supplementary Table 2). However, it is important to note that this effect is purely theoretical, as it requires a space length far from what is realised in hyphal cells. Moreover, this "diffusion-like" effect is not identical with the described AD of POs. AD is characterised by short-range motion of POs, enhanced by EE collisions, which is random and in our MSD analysis described by α~1. In contrast, individual uni-directional runs of POs, lasting for ~6.5 μm (Supplementary Table 2), during directed transport is characterised by α ~2, and thus is not diffusive.

Model validation for mutant cells (Δ*hok1*)

For the Δ*hok1* mutant cells, we measured the axial diffusion rate as $D = 0.0034 \pm 0.00002\ s^{-1}$ from $MSD(t)$ analysis. Using the drift velocity shown in Supplementary Table 2 and measured axial diffusion rate, and assuming the same background intensity as in *Δkin3* mutant, the model (S0) predicted the intensity profile for POs in the Δ*hok1* mutant cells as shown in Fig. 5c.

Hypothetical scenarios: model predictions

We used the model to explore the hypothetical scenarios shown in Supplementary Table 3 some of which were currently unable to be explored experimentally. The model allows us to examine the importance of the various processes in achieving an

even distribution of POs along the cell as well as the speed of mixing for these scenarios. The speed of mixing was characterized using the first arrival time of a PO to a distance distal from the hyphal tip. This was estimated using simulations of PO motility starting at the hyphal tip, averaged over a number of simulations (n=100-2000); random motility of individual POs along cell axis was simulated according to the parameters in Supplementary Table 3 for each scenario. To visualize the mixing process in a virtual hyphal-like cell (a cylinder of $10\ \mu m$ length and $2\ \mu m$ diameter), we extend the model describing the motility of POs with the inclusion of homogeneous lateral diffusion in the cell cross section and visualize the motility in the longitudinal projection.

Tracking LDs and POs in *U. maydis* and COS-7 cells

Image series of GFP-SKL expressing *U. maydis* and COS-7 cells, covering 15-45s observation time were used to automatically record PO (or LD) oscillations over time using a hosted image processing platform (https://apollo.ex.ac.uk/impi; for access to this platform contact J. Metz@exeter.ac.uk). Each image was filtered using a scale-space Laplace of Gaussian filtering approaching[8] over scales corresponding to the size range of POs. After filtering, a threshold was determined using the median absolute deviation as a robust estimator of the background level[9] and applied to the filter response to determine PO positions. Once detected, POs were tracked using the Jonker-Volgenant algorithm[10] on object positions to assign identities in successive frames. Results from this method were manually verified for accuracy.

Supplementary References

1. Bielska, E. *et al.* Hook is an adapter that coordinates kinesin-3 and dynein cargo attachment on early endosomes. *J Cell Biol* **204**, 989-1007 (2014).
2. Guimaraes, S. C. *et al.* Peroxisomes, lipid droplets and endoplasmic reticulum "hitch-hike" on motile early endosomes *J. Cell Biol.* **211**, 945-954 (2015).
3. Elgeti, J. & Gerhard G. "Wall accumulation of self-propelled spheres." Europhysics Letters **101,** 48003 (2013).
4. Yang, X. *et al.* Aggregation and segregation of confined active particles. Soft matter **10**. 6477-6484 (2014).
5. Schuster, M. *et al.* Controlled and stochastic retention concentrates dynein at microtubule ends to keep endosomes on track. *EMBO J* **30**, 652-664 (2011).
6. Schuster, M. *et al.* Kinesin-3 and dynein cooperate in long-range retrograde endosome motility along a nonuniform microtubule array. *Mol Biol Cell* **22**, 3645-3657 (2011).
7. Müller et al., Bidirectional transport by molecular motors: Enhanced processivity and response to external forces. *Biophys J.* 98, 2610–2618 (2010).
8. Lindeberg, T. *Lindeberg scale-space theory in computer vision.* (Kluwer Academic Publishers, 1994).
9. Murtagh, F. & Starck, J. L. Image processing through multiscale analysis and measurement noise modeling. *Stat Comput.* **10**, 95-103 (2000).
10. Jonker, R. & Volgenant, A. C., 325¬340 (1987). A shortest augmenting path algorithm for dense and sparse linear assignment problems. *Computing* **38**, 325-340 (1987).
